# Task-Specific Response Strategy Selection on the Basis of Recent Training Experience

**DOI:** 10.1371/journal.pcbi.1003425

**Published:** 2014-01-02

**Authors:** Jacqueline M. Fulvio, C. Shawn Green, Paul R. Schrater

**Affiliations:** 1 Department of Psychology, University of Wisconsin-Madison, Madison, Wisconsin, United States of America; 2 Department of Psychology, University of Minnesota, Minneapolis, Minnesota, United States of America; 3 Center for Cognitive Sciences, University of Minnesota, Minneapolis, Minnesota, United States of America; Philipps-University Marburg, Germany

## Abstract

The goal of training is to produce learning for a range of activities that are typically more general than the training task itself. Despite a century of research, predicting the scope of learning from the content of training has proven extremely difficult, with the same task producing narrowly focused learning strategies in some cases and broadly scoped learning strategies in others. Here we test the hypothesis that human subjects will prefer a decision strategy that maximizes performance and reduces uncertainty given the demands of the training task and that the strategy chosen will then predict the extent to which learning is transferable. To test this hypothesis, we trained subjects on a moving dot extrapolation task that makes distinct predictions for two types of learning strategy: a narrow model-free strategy that learns an input-output mapping for training stimuli, and a general model-based strategy that utilizes humans' default predictive model for a class of trajectories. When the number of distinct training trajectories is low, we predict better performance for the mapping strategy, but as the number increases, a predictive model is increasingly favored. Consonant with predictions, subject extrapolations for test trajectories were consistent with using a mapping strategy when trained on a small number of training trajectories and a predictive model when trained on a larger number. The general framework developed here can thus be useful both in interpreting previous patterns of task-specific versus task-general learning, as well as in building future training paradigms with certain desired outcomes.

## Author Summary

Predicting what humans will learn from a training task, in particular, whether learning will generalize beyond the specifics of the given experience, is of both significant practical and theoretical interest. However, a principled understanding of the relationship between training conditions and learning generalization remains elusive. In this paper, we develop a computational framework for predicting which of two basic decision-making strategies will be utilized by human subjects - 1) simple stimulus-response mappings or 2) predictive models. Through simulation, we show that the nature of the training experience determines which of these categories leads to better in-task performance; repetitive training on a small set of examples favors simple stimulus-response mappings, whereas training on a large set of examples favors predictive strategies. We then show that humans trained under these various conditions do indeed utilize the predicted strategy. Finally, we show that the strategies that are utilized during training predict generalization of learning. Those who learn simple mappings fail to generalize their new skills, in contrast to those who use default predictive strategies. The framework developed here is useful both in interpreting previous patterns of learning, as well as in building training paradigms with given desired outcomes.

## Introduction

One of the core problems in learning is determining the range of tasks and circumstances that a training paradigm will impact. Training can produce both learning that generalizes to new tasks and circumstances and learning that is restricted to the exact training conditions. The difficulty is that there are many paths to good performance in a given task – from more demanding routes in which extensive knowledge is acquired, to special purpose shortcuts that allow good performance with restricted knowledge. Without knowing which of the myriad possible approaches the subject will take during learning, there is no way to predict the generality of the eventual learning.

**Figure 1:**
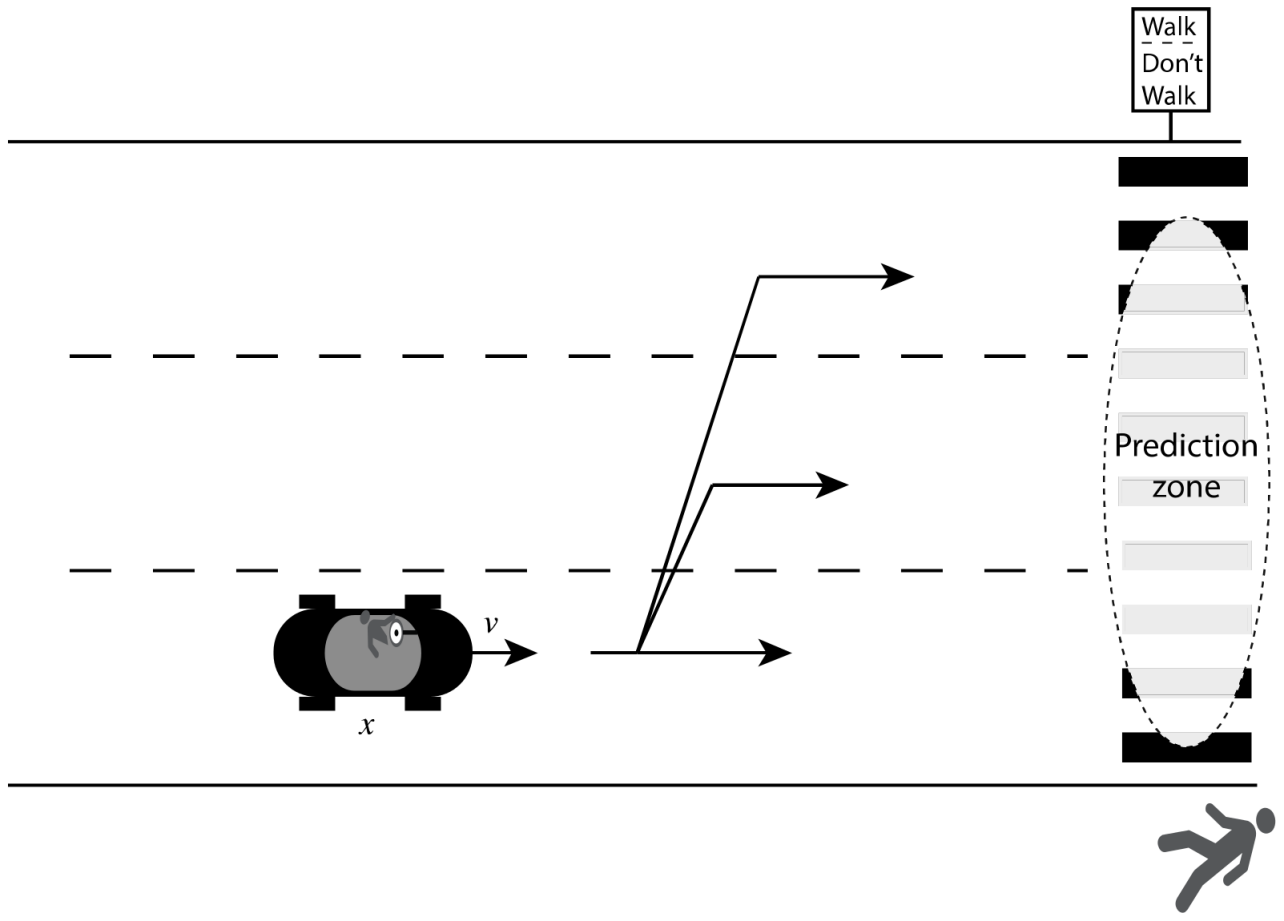
Many daily tasks can require prediction. When attempting to determine whether it is safe to cross the road, one must estimate the probability that approaching cars will be in the crosswalk at the same time as you. The uncertainty associated with this look ahead depends on both your estimate of the current state of the car - including its present position and velocity - and your estimate of its likely progression as it approaches the crosswalk - including possible lane changes. Errors in these estimates propagate with time, resulting in considerable uncertainty about the car's most likely future position. Both the cognitive demands of the prediction task and the error rate can be reduced using a simpler mapping strategy that entails crossing only when the walk sign is lit.

Even mundane tasks like learning when it is safe to cross the street have both narrow and general solutions. A general, and more difficult path, requires learning internal models for car and subject motion that can be used to look-ahead and predict future locations. To avoid being hit, you need to estimate the distance, speed, and direction of nearby cars and then to mentally simulate the cars' path through time. A similar simulation must also be run forward for your own progression across the street. The two simulations must be merged to determine if you are likely to be occupying the same physical space as a car at the same point in time (which we do not advise). While in general this works for any configuration of cars and pedestrians, such general strategies usually come with performance costs. Indeed, the sheer number of pedestrians struck by cars suggests that this type of look ahead is indeed both cognitively demanding and subject to error [1]. Even when not hit by a car, prediction errors can make us hurry a bit more than expected. Such errors are simply an intrinsic part of every task that requires look-ahead. Each time a prediction is made about a future state, some uncertainty is necessarily associated with that prediction (**Figure 1**). The further ahead you are asked to predict, the larger the accompanying uncertainty term becomes and the more frequent and larger will be the prediction errors you make [2]. One route to learning is improving this look-ahead process. This may be accomplished by enhancing the ability to estimate the initial state of the cars (distance/velocity) or by honing the internal model that is used to predict the progression of the cars (e.g., to gain more accurate knowledge of possible lane changes). Critically, any solution in this family will result in a reasonably general improvement in the probability of successfully crossing the street. Honing the internal model will result in benefits that do not depend on the exact set of cars, positions, or paths.

However, there is an alternative route to successful street crossing that does not require using (or improving) an internal model. Prediction can be eliminated by learning to map cues in the environment directly onto responses. The strategy of finding a mapping between perceptual information and actions that bypasses prediction is termed a *policy* in computer science. To illustrate, a simple policy for street crossing is to wait until the walk sign turns green. Policies greatly decrease both the cognitive overhead (thus freeing resources for other endeavors) and the error rate associated with predicting car trajectories. However, efficiency comes at a cost of inflexibility - policies are usually narrow in scope. The benefits of using a policy often completely disappear when various contextual changes are made to the environment (the most obvious being if no crosswalk sign is present, if the crosswalk sign is malfunctioning or unintelligible, etc.). We emphasize that response policies are not the same as response biases. Response biases correspond to systematic errors in performance, and any number of strategies may give rise to this behavior. Response policies are specific action selection strategies that rely upon learned associations between stimuli and responses as described above.

As there are clearly situations where humans do employ predictive models [3], but also situations where human behavior is consistent with inflexible mappings characteristic of using response policies, is it possible to predict when one strategy will be favored over the other? To address this question, we began with a simple hypothesis: namely that humans will employ the strategy that maximizes overall performance. If the demands of the task make response policies complex and difficult to learn, we predict that subjects will utilize predictive methods. Conversely, if the task demands make learning policies reasonably simple, we predict that subjects will eschew the computational costs of prediction in favor of exploiting simple mappings.

To test our hypothesis, we chose to utilize a task in which human subjects have been shown repeatedly to rely upon a predictive model: visual extrapolation through occlusion. Thus, for the purpose of this study, we have narrowed the focus to identifying the conditions under which subjects learn to adopt a simpler policy-based strategy that abandons predictive extrapolation in favor of a direct mapping between particular stimulus inputs and specific extrapolation endpoints. The extrapolation task we employed was selected because it meets several key criteria. First, we needed a task dimension, in this case training set variability, that could be manipulated to either strongly favor a predictive model or a policy-based strategy. We show that extensive repetition of a small set of stimulus-response pairs should strongly favor learning mappings, while fewer repetitions of a large set of stimulus-response pairs should favor continued use of a predictive model. The second criterion is that the task allows subjects to freely use either type of strategy when selecting responses. Finally, and perhaps most critically, the relative efficacy of both strategies needs to be independently estimated. To this end, we created a motion version of a well-studied extrapolation paradigm (e.g., [4]–[5]).

In the context of this task, we assess the use of two different subject-adopted strategies: one that is model-based and one that relies upon categorization-based stimulus-response mapping. In two experiments, we provide clear evidence that the strategy chosen by subjects, and thus the resulting generality/specificity, can be predicted by computing the best strategy given the training set they experience. The framework we develop here allows us to successfully make an explicit *a priori* determination as to which strategy would be most effective under what conditions and with clear predictions regarding the behavioral outcomes that would be observed in two experimental groups trained on two different sized sets of stimulus-response pairs as described below. The framework also provides a satisfying and parsimonious explanation for the question of “why” subjects select a given learning route – they simply choose the route that maximizes performance on the given task.

## Results

**Figure 2:**
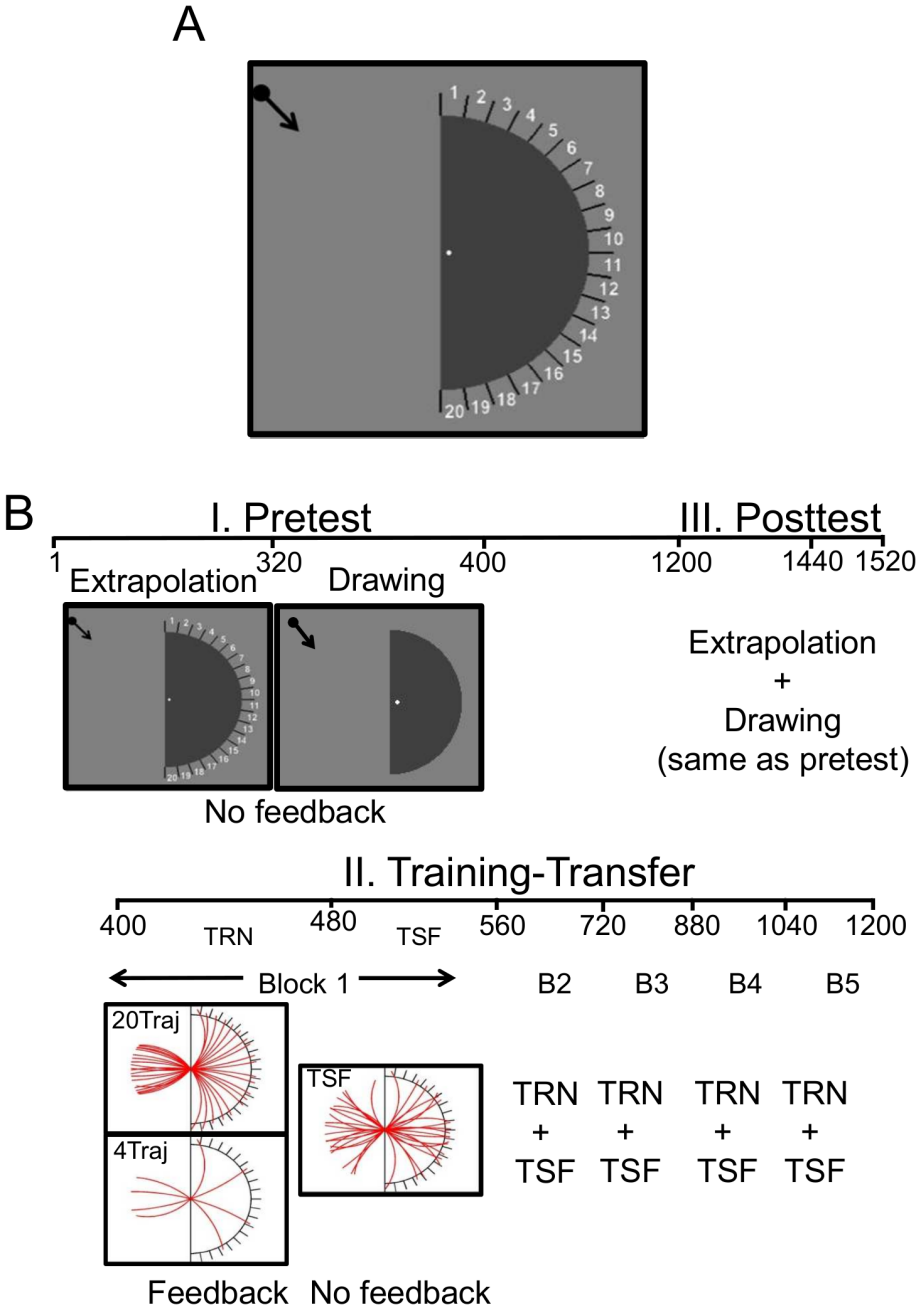
Experimental details. **A.** Schematic of experimental display. A dot follows a circular arc trajectory towards the midpoint of a half disk occluder and disappears while subjects fixate the small white dot. Subjects then mouseclick the bin they believe the dot will reemerge into. **B.** Schematic of the experimental sequence. Day 1 - An initial no feedback pre-test session of 320 trials with trajectories of random curvature and orientation and 80 trials of the drawing. Day 2: a training-transfer session consisting of five blocks each comprising 80 training trials followed by 80 transfer trials. During training trials, subjects were either trained to select bins for a set of 20 specific trajectories (‘20Traj’) or a set of 4 specific trajectories (‘4Traj’); feedback was provided. During the transfer trials, subjects selected bins for a set of 20 trajectories containing the 4Traj training set plus non-trained trajectories; feedback was not provided. Day 3: subjects completed a post-test session that was identical to the pre-test session. Note that the trajectory set used during the drawing generation task was identical to the transfer (‘TSF’) set.

In Experiment 1, subjects watched a dot travel along a circular trajectory before disappearing behind a stationary occluder (see **Figure 2A**). After the dot's disappearance, subjects were asked to choose which of 20 “bins” (each with a span of 9°) residing at the opposite edge of the occluder the dot would reemerge within. The bin size was chosen to approximate subjects' inherent reliability in the extrapolation of curved contours using a similar stimulus [4]–[5]. The basic design consisted of a pre-test, extrapolation training with different set sizes, followed by a post-test to determine the impact of training (**Figure 2B**, see Methods for a full description). During both pre-test and post-test, circular trajectories were drawn randomly from the full 2D space of curvature and orientation and no feedback was given so as to discourage learning. Subjects also completed a trajectory generation task in which they were shown the same moving dot stimulus, but after the dot reached the occluder the subjects were asked to use the mouse to draw the remainder of the trajectory. The ability to perform this type of data generation task is strongly indicative of having a predictive model.

**Figure 3:**
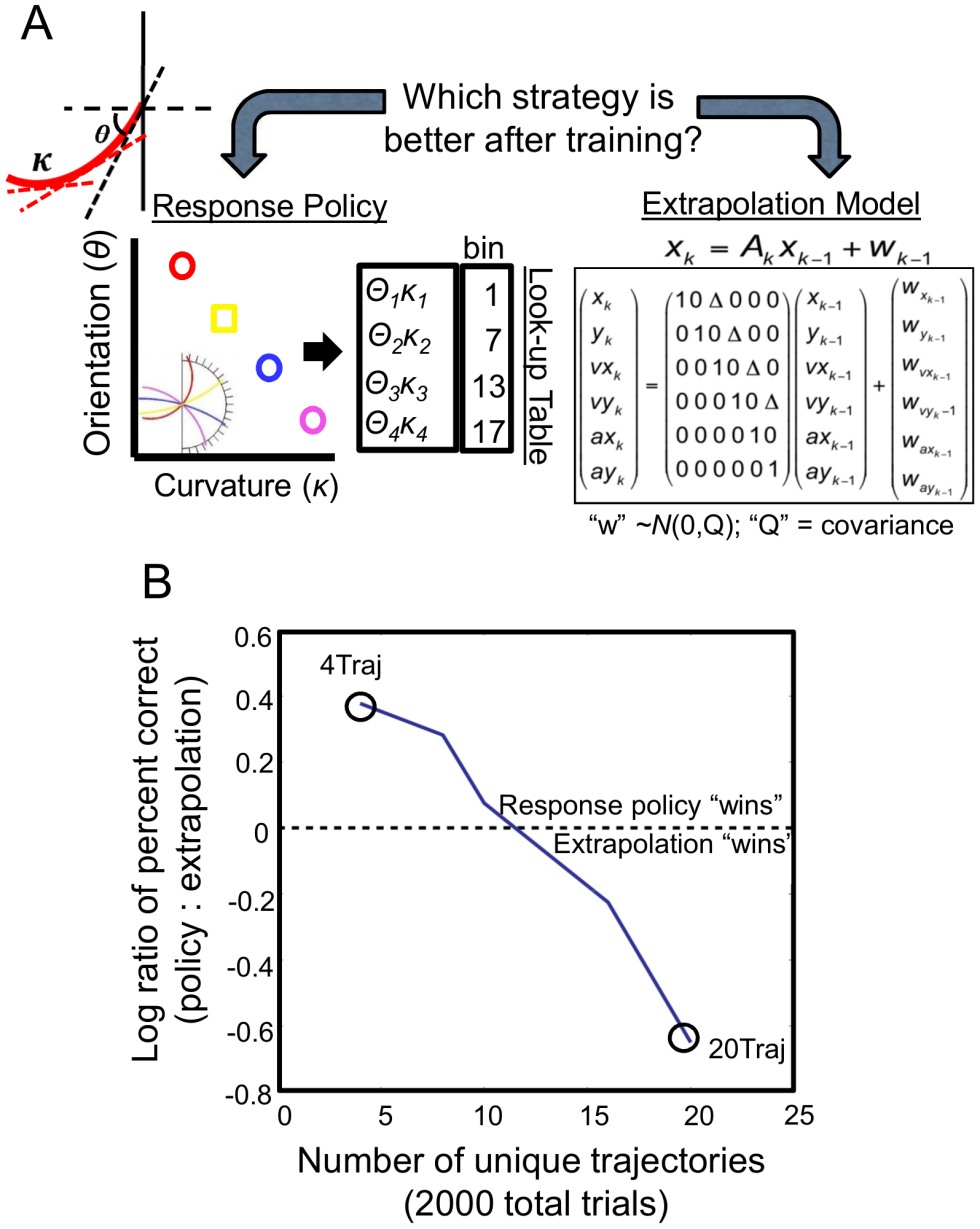
Simulation details. **A.** Illustration of response mapping and predictive model strategies. Subjects may learn response policies that map trajectories with specific combinations of curvature and orientation to specific bins, represented by a look-up table. Alternatively, they may use an extrapolation model that estimates the curvature and orientation of the visible portion of the trajectory and extends it through the occlusion region towards the bins. **B.** Simulations of these strategies (see text for details) predicts that a response policy strategy outperforms predictive model strategies for small sets of training trajectories but becomes dominated as the number of training trials increases. This predicts 4Traj -trained subjects would adopt a response policy strategy after training while 20Traj -trained subjects would rely on extrapolation models in order to make their bin selections.

During training, subjects were provided feedback on their performance. These trials comprised a subset of fixed trajectories, which were repeated (in pseduorandom order) for a total of 200 exposures. We picked two training set sizes, “small” and “large”, with the former being predicted to favor mappings and the latter predictive models. We generated predictions by simulating performance by both a forward predictive model (a Kalman filter) that provides excellent fits to pre-test data and an agent that learns direct mappings between the visible trajectory statistics and response bins (a model-free Q-learner; see **Figure 3A**, Methods and **Text S1** and **Text S2** of the Supporting Information for details). The simulations allowed us to make clear and quantitative *a priori* predictions about the effect of training set size on the performance of a predictive model strategy versus a policy-based strategy. This analysis suggested that a training set of 4 trajectory-response pairs would favor learning a response policy, while a training set of 20 trajectory-response pairs would favor the predictive model strategy, with the total number of training trials equated across groups. These values were used in the experiment. Subjects were randomly assigned to either a group that was trained on only 4 of the set of possible trajectories with a corresponding 4-bin response space (4Traj), or a second group that was trained on 20 different trajectories corresponding to the full 20-bin response space (20Traj; see **Figure 3B**). Both groups completed 5 training blocks of 80 trials each (see Methods for more details). Therefore, within these blocks, either four trajectories were presented 20 times per block (in the 4Traj group) or 20 trajectories were each presented four times each (in the 20Traj group). The four trajectories presented to the 4Traj group were a subset of the twenty trajectories presented to the 20Ttraj group.

To assess the effects of training, both groups underwent a post-test identical to the pre-test (both the choice and generation tasks). Recall that the post-test comprised a set of no-feedback trials using trajectories that differed from the training sets. Our prediction was that the 4Traj group would transition away from using a predictive model and toward using a simple mapping strategy. This should manifest in a number of ways. First, during training we expected to see substantial gains in accuracy in the 4Traj group (far exceeding what would be possible using a predictive model). In fact, given the perceptual separation between the 4Traj trajectories, we expected performance during training to approach ceiling levels. However, at post-test, we expected that the use of the same (now inappropriate) mapping strategy would result in markedly poorer performance than was seen at pre-test. Again, this trade-off reflects the very heart of learning a response policy. Bypassing the need for a predictive model allows for extremely accurate responses to be made on training stimuli, but provides for no flexibility to deal with stimuli not in the training set. Conversely, we predicted that the 20Traj–trained subjects would not change their strategy from pre-test to post-test. In the 20Traj group, we predicted only modest improvements in performance at best as the number of training trials is theoretically unlikely to dramatically improve predictive performance via either changes in the state estimate or the internal trajectory model.

**Figure 4:**
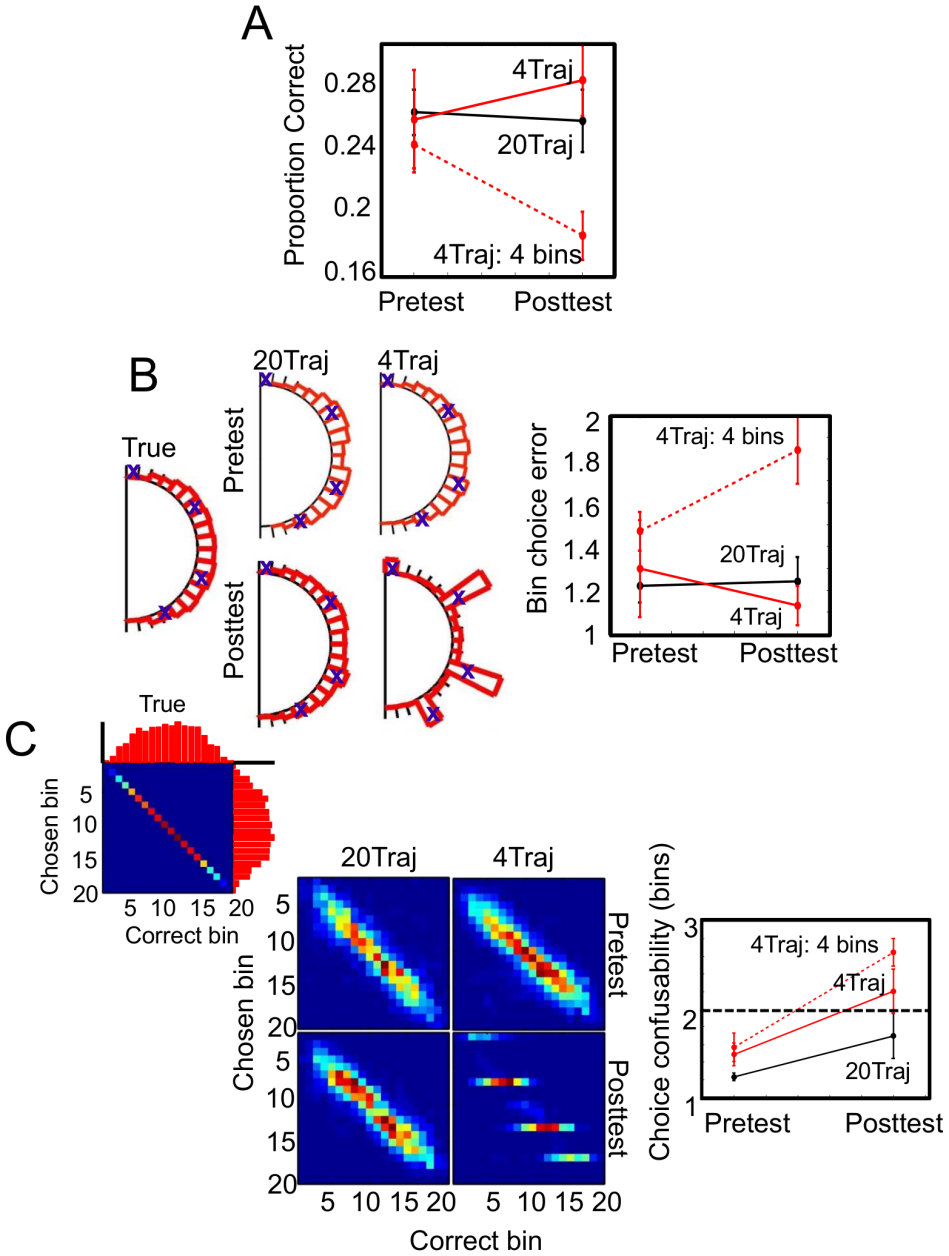
Experiment 1 results. **A.** Bin choice accuracy. The line graph depicts mean proportion correct +/−1 SEM for the 20Traj and 4Traj groups. Both groups performed similarly and above chance (0.05) at pre-test. At post-test, the majority of the 4Traj group (7/10; dashed line) continued to use the four trained bins. This resulted in a significant decline in accuracy. By contrast, the remaining 4Traj group subjects who returned to using the full set of response bins (3/10; solid red line) along with the 20Traj resulted in equivalent performance. **B.** Bin choice histograms and corresponding error. Both groups distributed their bin choices in a matter similar to the true distribution at pre-test. During post-test, the 20Traj group along with a subset of the 4Traj group (3/10) continued to distribute their choices in this way, whereas the remaining majority of the 4Traj group (7/10) changed their choice behavior, primarily selecting the bins associated with the four trajectories during training. These strategies were associated with differences in amount of error. In particular, subjects who distributed their choices at pre- and post-test were little more than 1 bin off from true on average. Those 4Traj subjects who continued to select the four trained bins primarily at post-test saw a decline in performance to nearly 2 bins off on average. **C.** Bin choice confusability. The far left panel represents how perfect performance would appear in the choice confusability matrix format – the chosen bin always perfectly matches the correct bin. There is more mass in the middle bins simply because there are more combinations of orientation and trajectory that terminate toward the middle of the arc than toward the ends. The middle panel shows that at pre-test both groups exhibited similar degrees of confusability in selecting appropriate bins for the trajectories observed. At post-test, this confusability was largely unchanged for the 20Traj group, whereas it increased for the 4Traj group who assigned the four trained bins to a broader range of trajectories. The line graph depicts mean confusability +/−1 SEM for the 20Traj, total 4Traj group, and a subset of the 4Traj group who primarily use only the four trained bins at post-test (see text).

There were no significant differences in pre-test behavior between the two groups in choice task accuracy (both groups ∼25% correct (chance performance = 5%), t(15) = 0.72, p = .49; see **Figure 4A**). On average, the 20Traj group had only a slight advantage with a mean absolute distance of 1.22 bins (+/−0.0753 SEM) from true over the 4Traj group's mean absolute distance of 1.42 bins (+/−0.0876; see **Figure 4B**). We also considered whether subjects were biased to under- or overshoot the true trajectories' endpoints by taking the signed mean distance where negative values correspond to a tendency to undershoot the endpoint (as if underestimating curvature). There was no systematic bias across subjects in either direction (M_20Traj_ = 0.0067+/−0.11 bins; M_4Traj_ = −0.16 +/−0.12) Additionally, Kolmogorov-Smirnov tests failed to reject the null hypothesis that the two group distributions did not differ from their respective ideal choice bin distributions (D = 0.0294, p = 0.1243 (4Traj) & D = 0.0281, p = 0.334 (20Traj) see **Figure 4B**). Finally, we considered subjects' bin choice confusability. An ideal subject accurately estimates the curvature and orientation of the visible portion of each trajectory and extrapolates to the correct bin. However, due to sensory noise, actual human subjects often misestimate the visible trajectories and extrapolate to the wrong bin. Sensory noise also causes different trajectories to be perceptually less discriminable and thus more confusable. We measure subjects' trajectory confusability by the range or number of bins around the true bin that the subject deems acceptable – the larger the range, the greater the confusability. At pre-test, there is no difference in confusability across the two groups (t(15) = 1.6201, p = 0.126; **Figure 4C**). Taken together, the pre-test results demonstrate that neither group had a pre-training bias toward disproportionately choosing specific bins.

The pre-test results also verify that subjects are employing predictive models to arrive at their choices. In the absence of continuous visible information (i.e., the impact of occlusion) and the requirement that a response be made later in time and space, subjects may either rely on an internal belief about where the dot is going (i.e., a model-based prediction) when choosing a bin, or they must pick one at random - our data clearly rule out the latter strategy. How subjects learn these predictive models is beyond the scope of this paper and will be addressed in future work.

The model simulations predicted that by the end of training, 4Traj subjects learning trajectory-to-bin mappings would achieve near ceiling performance. This is a significant advantage over what could be expected by relying on a predictive strategy (approximately 1.5 times better, see **Figure 3A**). Indeed, as predicted, the 4Traj group choice accuracy improved significantly to reach 87.3% on average by the final training block (+/−2% SEM; F(1,9) = 738.04, p<0.001). The accuracy of the 20Traj group exhibited modest, but nevertheless significant improvements during training, with accuracy climbing to 35.2% on average by the final training block (+/−3% SEM; F(1,6) = 10.85, p = 0.0165).

Thus, both groups were able to use the feedback during training to improve their performance. The critical test of our primary hypothesis, however, was the strategy each training group adopted during the post-test session. In direct contrast to pre-test, clear differences in choice behavior were evident between the two groups. As depicted in **Figure 4A**, the accuracy of the 20Traj group remained stable as compared to pre-test performance (F(1,6) = 0.17, p = 0.69), a result consistent with continued use of the same type of response strategy. We note that although the 20Traj group accuracy remained stable, the choice distributions became even better aligned with the ideal (‘true’) distributions at post-test relative to pre-test as revealed by a smaller computed distance using the same Kolmogorov-Smirnov test (D = 0.0246, p = 0.5045). Thus, although we did not expect a strategy shift or a drastic improvement in performance, the 20Traj training did provide modest benefit.

By contrast, the accuracy of the 4Traj group showed a decline in accuracy from pre-test to post-test. At the group level, this decline was not significant (F(1,9) = 2.59, p = 0.1423); however, as will be discussed in detail next, seven out of ten subjects continued to rely almost exclusively on the four trained bins at post-test while the remaining three subjects returned to using the full set of bins. The former group showed a significant decline in choice accuracy relative to their pre-test performance (F(1,6) = 7.21, p = 0.036) whereas the latter group showed no change in accuracy, like their 20Traj counterparts (F(1,3) = 0.81, p = 0.463; **Figure 4A**). The decrease in accuracy for these subjects is accompanied by an increase in mean absolute distance between their bin choices and the true bins (see **Figure 4B**).

The decline in performance seen in the majority of 4Traj subjects is consistent with the predicted impact of learning to utilize an inflexible policy to make decisions. Moreover, the mapping strategy should rely on the use of the same bin whenever the input is deemed the same as one of the training inputs. As is evident in **Figures 4B & 4c**, there was a large increase in the probability of 4Traj subjects selecting one of the trained bins associated with a significant increase in confusability (F(1,6) = 30.91,p = 0.0014) for those subjects. That is, subjects were willing to accept the four trained bins as appropriate responses for a much broader range of trajectories than they were at pre-test.

This was a pattern that emerged early in training – being evident even in the first no-feedback transfer block – and remained throughout in the majority (7/10) of 4Traj subjects as described (**Figure S1**). We note that the key difference between the transfer and post-test trials is that the post-test trials were randomly sampled from the full space of possible trajectories, as opposed to the fixed set of transfer block trajectories, which contained both trained and untrained trajectories. Further analysis of the transfer block data revealed that the 4Traj-trained subject bin choices were significantly more accurate for trained trajectories versus untrained trajectories (t(18) = −4.5736, p<0.001). This is due largely to the fact that the majority of these subjects continue to use the four trained bins for all trajectories during these blocks (see **Figure S1**). The 20Traj subjects also exhibit a small but non-significant advantage for trained trajectories (t(18) = −1.8681, p>0.05). Generally, the 20Traj group's use of a prediction-based strategy provides them with a performance advantage in comparison with their 4Traj-trained counterparts who rely on a categorization-based strategy for untrained trajectories (see **Figure S2**).

**Figure 5:**
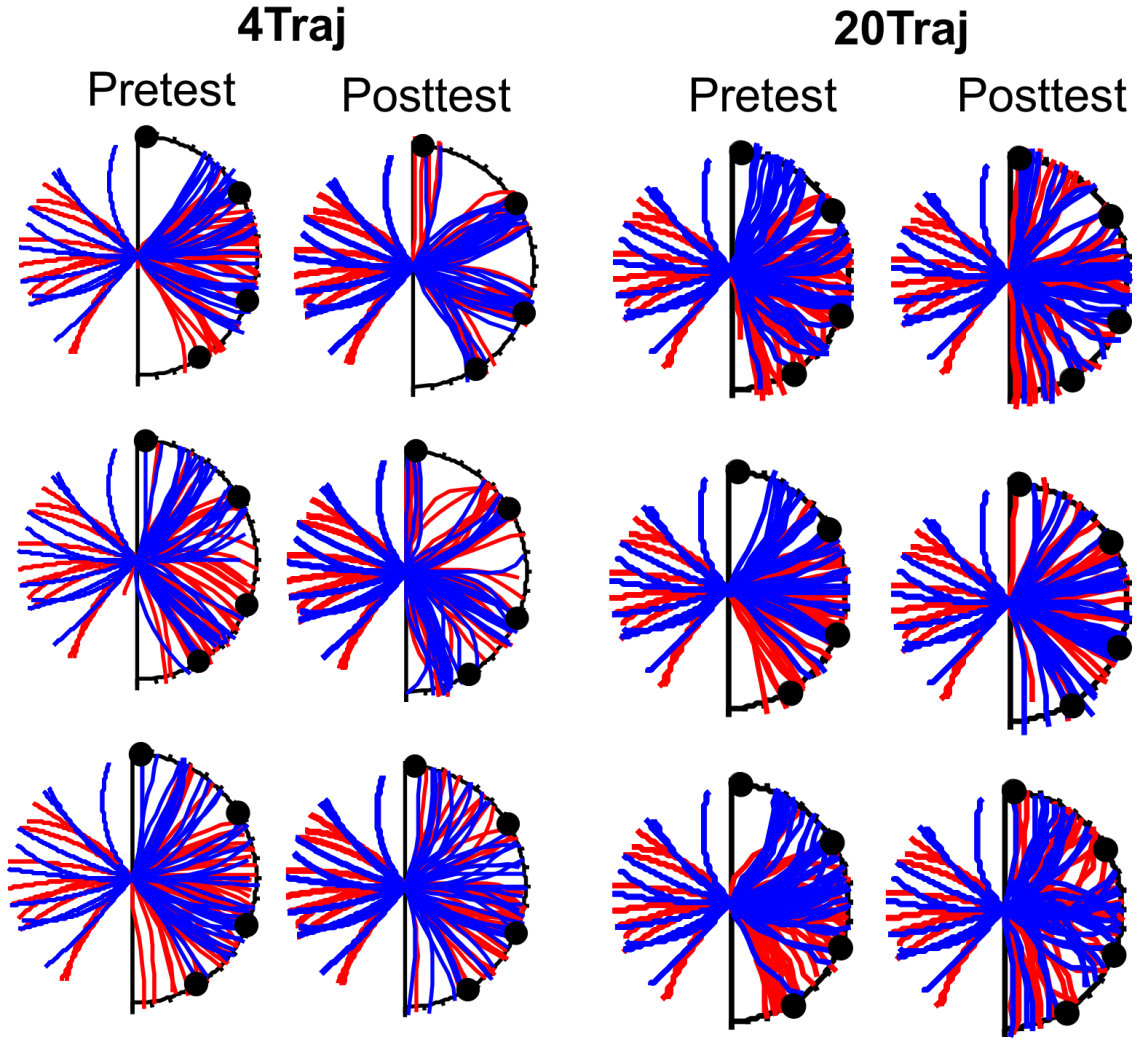
Experiment 1 drawing task results. Sample smooth drawing data for three 4Traj-trained subjects in pre-test and post-test (two left-most columns). The four black dots correspond to the bins associated with the 4Traj training trajectories. Visible trajectories of negative curvature (i.e., concave downward) are depicted in red, while those of positive curvature (i.e., concave upward) are depicted in blue. Thus, if the subject properly continues the trajectories, red drawn curves should approach the bins through the occlusion region from the left and blue drawn curves should approach the bins from the right. The upper and middle subjects persist in using the four trained bins at post-test during the bin choice task, whereas the bottom subject appears to use a predictive extrapolation strategy during the bin choice task. Comparison of their drawn trajectories reveals variable degrees of performance overall, with a tendency to ensure the drawn trajectories end towards the four trained bin locations congruent with the tendency to favor those bins during the choice task. Ensuring the drawn trajectories end towards the four trained bin locations is in conflict with accurately continuing the visible trajectory as revealed by increased numbers of curvature sign reversals (e.g., extending a concave upward trajectory with a concave downward one) and sharp orientation changes. For comparison, the drawn trajectories for three sample 20Traj-trained subjects are depicted in the final two columns with the same format. All individual subject data for both groups can be found in **Figure S3**.

Finally, subject data on the generation task, in which subjects drew the extended trajectories of the transfer trajectory set, show a qualitative change in behavior after 4Traj training. The plots in **Figure 5** depict the sets of trajectories drawn by three 4Traj subjects at pre-test and post-test (the first two columns; see **Figure S3** for the remaining subjects' trajectories). For the purpose of comparison, the bins from the bin choice task have been included along the curved edge of the occluder with very small tickmarks, and the black dots correspond to the four trained bins. The true trajectory endpoint distribution is depicted by the transfer set ‘TSF’ in Figure 2B, which we note does not span the full edge of the occluder. Of particular note is that 4Traj-trained subjects who used only 4 bins in the choice task during transfer and post-test showed a similar trend in the drawing data. That the drawn trajectories show no resemblance to a circular trajectory demonstrates that they were not using their previous predictive model, but instead were aligning their drawn trajectories with one of the four trained endpoint locations. Given that the drawing trajectory set included the 4Traj training trajectories, we analyzed the data by removing these trajectories and looking at performance on the remaining trials. When we compare the distributions of the subjects' drawn endpoints with those of the true trajectory for these diagnostic trials, we find that no 20Traj subjects deviate from true at post-test (Kolmogorov-Smirnov tests, p>0.05; see the drawn trajectories for the three sample 20Traj subjects depicted in the final two columns of **Figure 5** and the remaining individual 20Traj subjects in **Figure S3**). By contrast, half of the 4Traj subjects deviate significantly from true at post-test (similar to the 7/10 subject count who continue to use the four trained bins during the post-test of the choice task). For validation purposes, none of the subjects in either group drew trajectories with endpoints that deviate from true for the trajectories that do terminate at the locations where the four trained bins occurred in the choice task.

**Figure 6:**
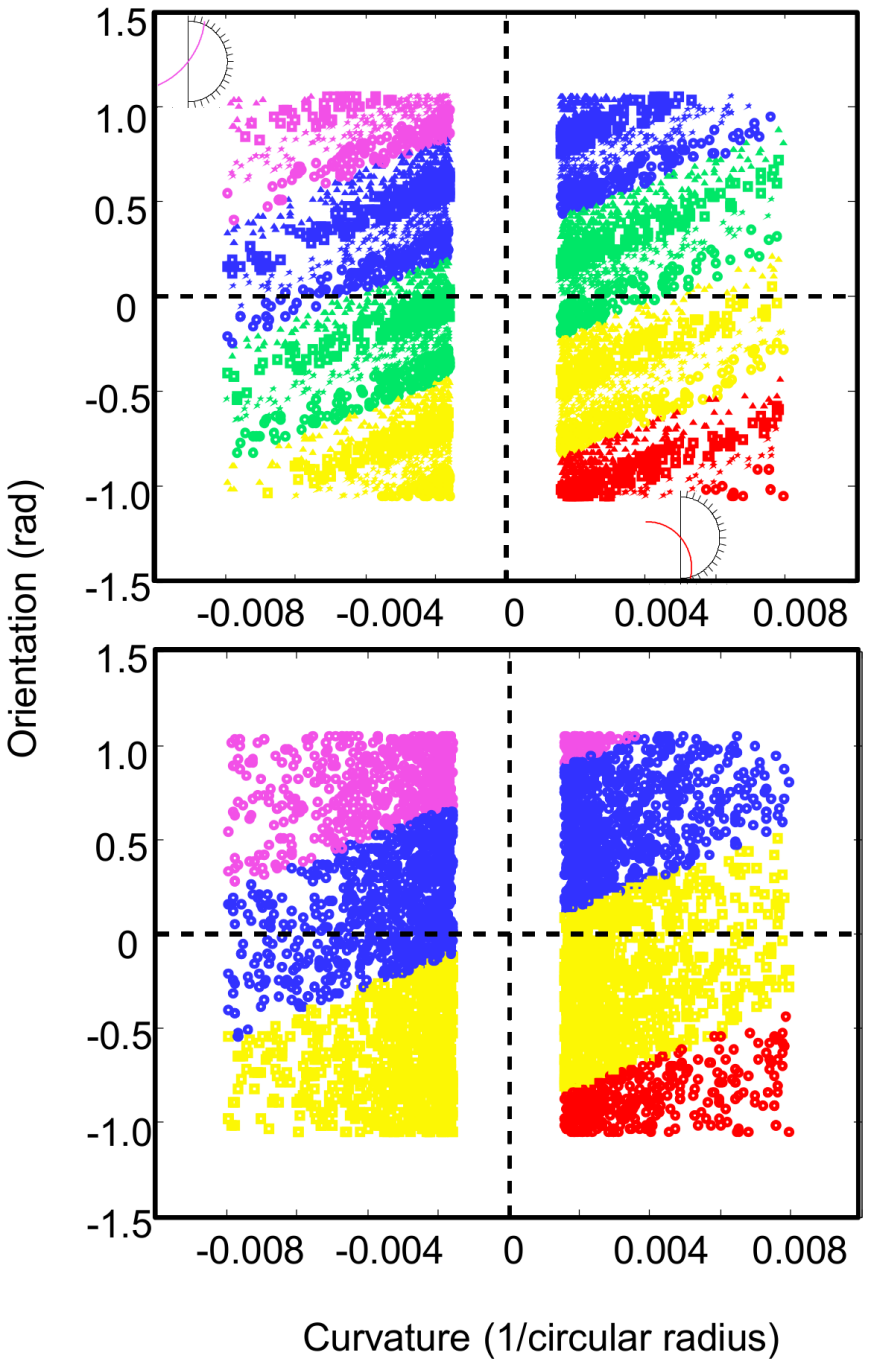
Bin categories within the 2D parameter space. Left: The natural 20-category partitioning of the 2D parameter space with each category depicted by one of 20 color + symbol combinations. For each bin, there is a distribution of trajectories with mean curvature and orientation that will reemerge in that bin. Note that the size of these distributions changes with the size of the bins. Here, we used bins of size = 9 deg. Right: The idealized 4-category partitioning of the parameter space based on an overgeneralization of the trained bins to the trajectory set using a ‘nearest bin’ classification. See main text and **Text S3** of the Supporting Information for more details. The gap in the parameter space around zero curvatures corresponds to the region omitted to avoid ‘illegal’ trajectories (see Methods for details).

**Figure 7:**
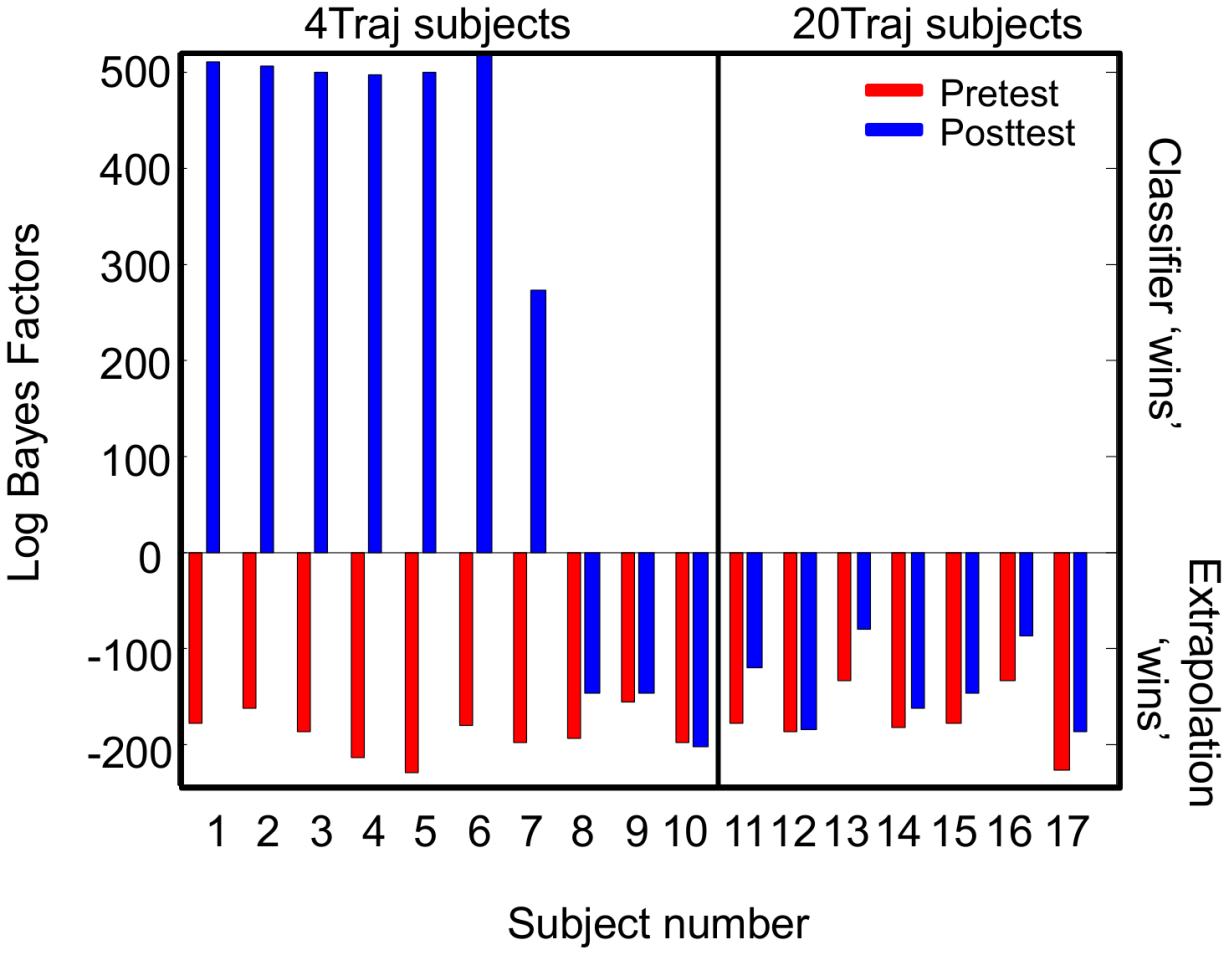
Subject bin choice distributions replotted within the 2D parameter space. Left column: The pre-test distributions for representative 20Traj (top) and 4Traj (bottom) subjects are nearly identical and consistent with the natural 20-category partitioning of the space. Right column: The post-test distributions show distinct patterns of performance. The 20Traj group (top) performance is largely unchanged as a result of training as seen in the subject's data shown here as well as in the remaining 20Traj subject data plotted in Figure S3A. The 4Traj group performance tends to favor the four trained bins. Here, the four symbols corresponding to the four trained bins (compare to the inset) correspond to nearly all of the plotted subject's choices. This same pattern is evident in the majority of the remaining individual 4Traj subject data plotted in Figure S3A. This four-bin response scheme is consistent with the idealized 4-category partitioning of the parameter space based on an overgeneralization of the trained bins to the trajectory set using a ‘nearest bin’ classification (recall Figure 5).

**Figure 8:**
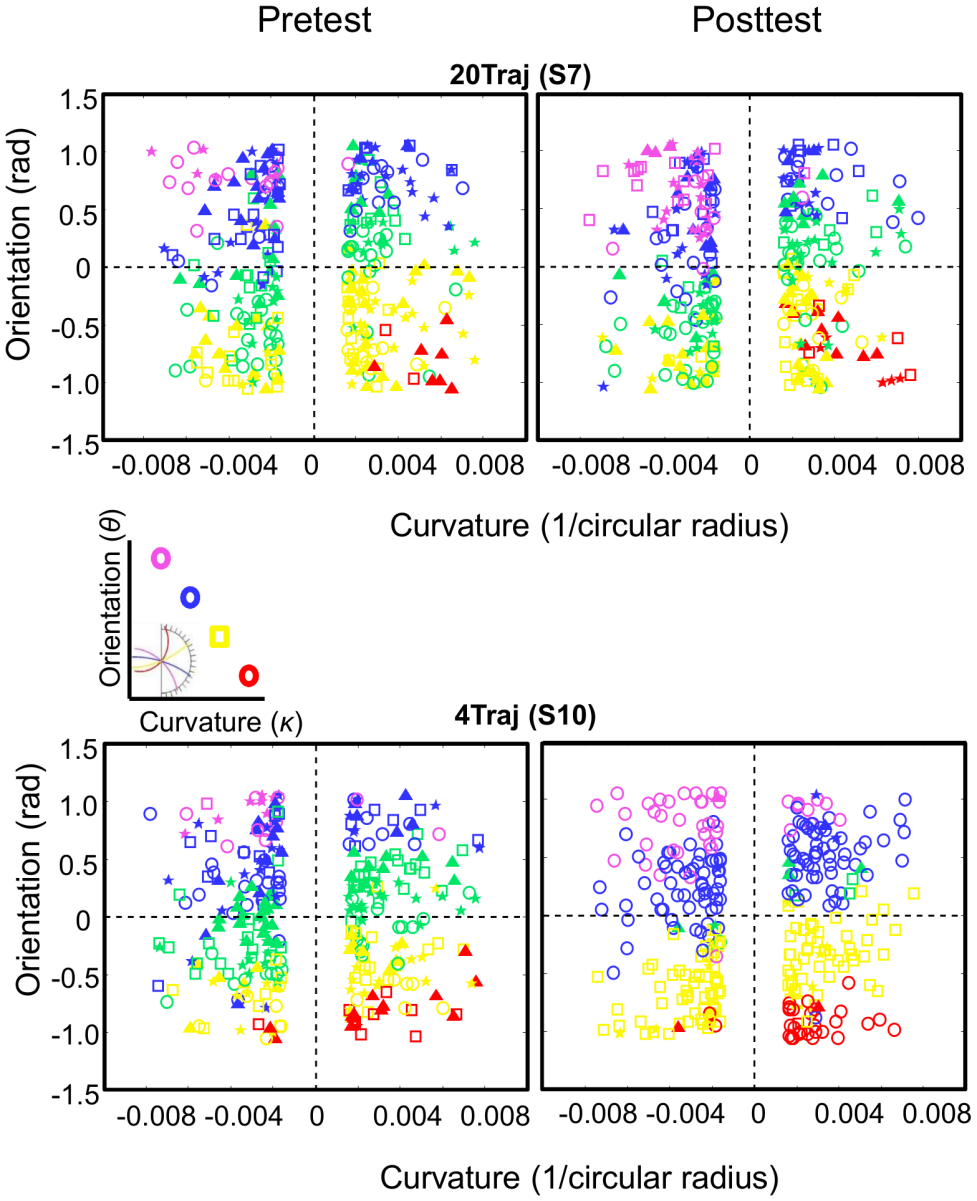
Bayes factors for all 17 subjects who participated in experiment 1. Red bars correspond to the (log) Bayes factors for pre-test and blue bars correspond to the (log) Bayes Factors for posttest. Positive values correspond to a better fit by the 4-category classifier; negative values correspond to a better fit by the full 20-category extrapolation model. As expected, all subject performance is better accounted for by the full 20-category extrapolation model. At post-test, 7 of the 10 4Traj subjects' data are better accounted for by the 4-category classifier. The performance of the remaining 3 4Traj subjects and all 20Traj subjects remains better accounted for by the full 20-category model. All Bayes factors are significant.

The post-test choices of the 4Traj group suggest that confusable inputs (i.e., similar trajectories) were mapped to the trained responses, resulting in a four-response classification. The rationale for this idea is that the pre-test data show clear evidence of the confusability of trajectories (recall **Figure 4**). The set of trajectories that map to a bin is well-modeled by a Gaussian distribution over trajectory orientation and curvature. Learning a mapping with input confusability is equivalent to learning to divide the 2D space of orientation and curvature into categories (responses). To assess the extent to which this strategy truly corresponded with subject behavior we simulated the performance of two types of decision makers. One simulated decision maker had an accurate understanding of the 20 trajectory parameter categories corresponding to the 20 response bins (i.e., could properly divide the 2D space of orientation and curvature into the 20 response bins), while the second divided the same space into 4 categories (see **Figure 6** and **Text S3** of the Supporting Information), comprising the sets of new trajectories that are confusable with the four trained trajectories. The subjects' bin choice distributions replotted in the 2D parameter space (**Figure 7** and **Figure S4**) demonstrates that the distribution of choices at pre-test (recall **Figure 4**) is consistent with 20-category based responses for both groups, though with some blurring of the categories. This is to be expected with non-perfect estimation and extrapolation. To quantify our qualitative observation, we then assessed the extent to which each of the two models correctly predicted individual subject choice behavior at both pre- and post-test. The Bayes factors of the four-category classifier model performance relative to the full model for each individual subject can be seen in **Figure 8**. At pre-test all subjects were better fit by the 20-category than the 4-category model as expected. However, at post-test, a majority of the 4Traj subjects were better fit by the 4-category model (**Figure 7** and **Figure S4**).

**Figure 9:**
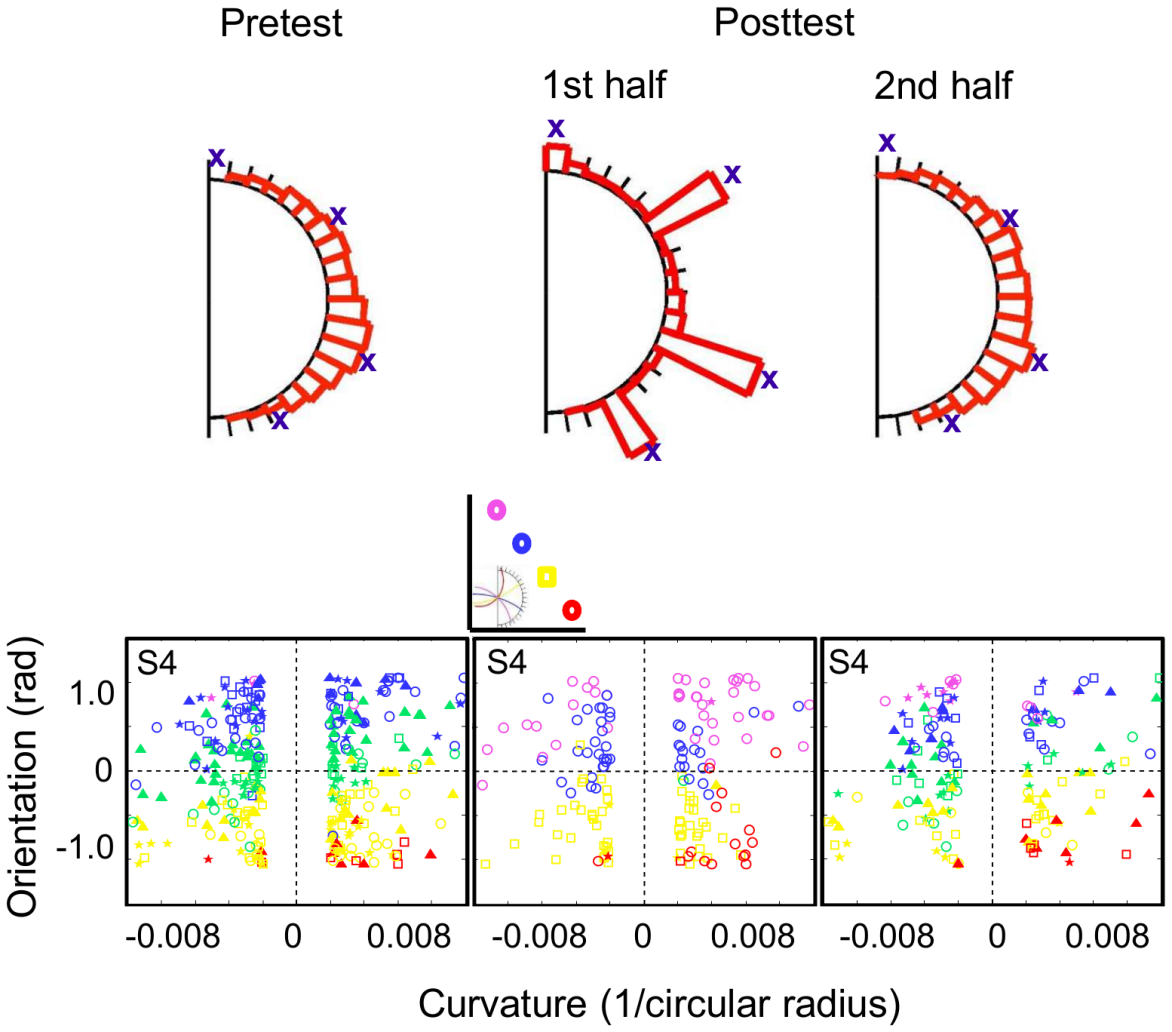
4Traj group receiving feedback halfway through post-test (n = 4). These subjects behave identically to the original 4Traj group at pre-test and during the first half of posttest (3 out of 4 exhibit classification behavior; see Figure S4 for remaining 3 subjects' choices). Once feedback is reinitiated during the second half of post-test, which signals that the predominant use of the four trained bins is no longer sufficient, the categorizing subjects rapidly transition back to an extrapolation strategy to improve their accuracy.

Finally, in order to demonstrate that the observed behavior was the result of a strategy shift in the service of maximizing performance, we established that the persistence of the 4-category response strategy into the transfer blocks was completely dependent on a lack of feedback (thus, the subject had no error signal that would indicate the strategy was not just as efficient as during the training). Indeed, if subjects were provided feedback on their performance with the post-test trajectories (which end in all 20 bins), it should be immediately apparent that the four category mapping strategy resulted in far larger errors than would be the case for a predictive model. Four additional subjects underwent the same 4Traj training as described above. The only difference was that feedback was provided for the second half of the post-test. As can be seen in **Figure 9**** and Figure S5**, the data from the pre-test and the first half of the post-test closely mirror the previous results with the subjects at pre-test responding in a manner consistent with the 20-category model and at post-test with the 4-category model (3/4 subjects). However, once feedback was provided, an immediate switch back to the 20-category model was observed. It is worth noting that this final behavior effectively rules out one additional alternative hypothesis – namely that the behavior of the 4Traj subjects could be the result of combining the results of a predictive model with a prior learned over bins. Because the subjects experienced correct responses at only four of the bins, the posterior over bins would thus also share that property. Since data accumulated into a prior converges to a delta function in the limit of infinite data assuming the identifiability of the sufficient statistics on x, the impact of additional data on the prior monotonically decreases as the amount of data goes to infinity. Thus, undoing this type of learned prior could only be accomplished via a significant amount of data, far less than appears to be required for them to completely alter their choice strategy after feedback is provided in the post-test. Instead, the behavior does indeed appear to be most consistent with a shift toward a pure categorization approach with no predictive model being utilized.

The results of Experiment 1 suggest that human subjects rely on contextual experience in order to determine the simplest strategy that maximizes task performance. In the context of trajectory extrapolation, all subjects initially relied upon a model-based extrapolation strategy that extends the visible trajectory through the occlusion region via a trajectory dynamics model, in this case, one that is consistent with a parameter space partitioned into 20 categories. However, a simpler classification strategy emerges with repetitive training on a limited set of trajectories that indicates to the subject that only a few specific responses are needed. This strategy remains the preferred one until new information is provided that the context has changed, upon which subjects rapidly switch strategies to improve performance and more effectively meet task demands.

However, it is not clear what kind of mapping the 4Traj strategy classification corresponds to. One possibility is that the mapping is between trajectories and response bins - subjects may learn that bins 1,7,13,&17 are the only appropriate responses in this experiment. Alternatively, subjects may learn a more general mapping between trajectories and regions of space, for example, the regions of space indicated by the predictive model for the trained stimuli. In other words, feedback might serve to crystalize or reinforce particular model extrapolations, rather than particular responses.

We tested these possibilities in a second experiment by having subjects make their bin choices at multiple distances from the point of occlusion (i.e., along the curved edge of multiple sized half-disk occluders) after training. If classifying subjects map inputs to response bins, this manipulation should have no effect on the persistent use of the identical four bins at post-test. If subjects map inputs to particular model extrapolations, a new set of four should emerge with each new occluder radius that is sensible for that prediction distance and set of trajectories experienced during training.

Predictions for each of the two mapping hypotheses are shown in Figure 9a, with response mapping represented by the blue dashed lines and extrapolation mapping represented by the green dashed lines. A third possibility is that subjects only learn a mapping for the specific occluder used in training—that is, only for the mid-sized. In this case, we expect choice behavior would revert to pre-test.

**Figure 10:**
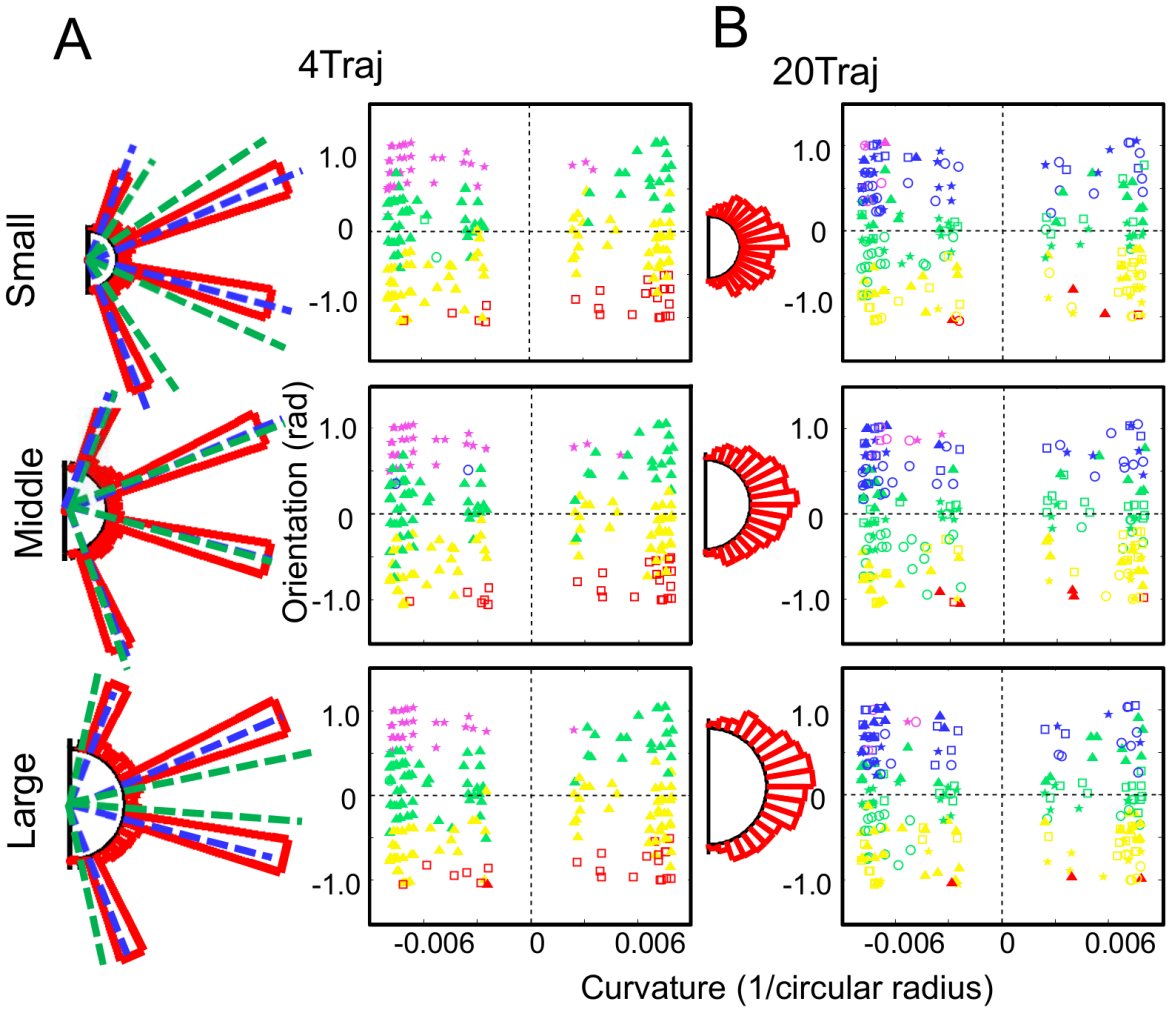
Post-test bin choice distributions for experiment 2. **A.** Choice histograms and bin choice parameter space plots. The blue dashed lines correspond to the predicted classifier strategy that uses the same four numbered bins regardless of occluder size. The green dashed lines correspond to the predicted classifier strategy that uses a logical four-bin set dependent upon occluder size. The red choice histograms correspond to the grouped subject data. The histograms reveal that the 4Traj group exhibits the same classifying behavior as in Experiment 1 for all three occluder radii, whereby the peaks in the choice histograms coincide with the blue dashed lines of the predicted classifier histogram. Additionally, the 4Traj subject choices (a representative subject, S10, is shown here while all are shown in **Figure S6**) are sensible given trajectory dynamics, revealing that subjects do in fact overgeneralize the four trained bins using them in a ‘nearest neighbor’ way as demonstrated by the same four color-symbol combinations in each of the three parameter space plots. This reveals subjects adopt the simplest strategy supported by their experience. **B.** Same format as **A.** The 20Traj group consistently performs in accordance with a predictive strategy throughout the experiment, as predicted and revealed by both the choice histograms and the bin choice parameter space plots (a representative subject, S7, is shown here while all are shown in **Figure S7**).

For subjects showing the response mapping strategy (7 out of 10) we found that they used the same response bins for all three occluder conditions (**Figure 10A**** and Figure S6**), strongly supporting the idea that subjects are learning a response mapping. Consistent with the results of Experiment 1 and the behavioral predictions, the 20Traj subjects consistently performed in accordance with the full model throughout the experiment (**Figure 10B**** and Figure S7**).

We conclude from these results that 4Traj subjects who adopt a classification response strategy at post-test use this strategy to bypass extrapolation altogether. If they were encoding a four-bin response strategy at a decision-level that runs the trajectory forward through occlusion (i.e., via extrapolation), a different set of four bins would be expected with each occluder condition because the trained trajectories reemerge in different bins for the different occluders. Instead, the categorizing subjects chose among the same four trained bins for all three occluder radii. This strategy saves computational cost as it eliminates using a forward predictive model.

## Discussion

In two experiments, we have provided clear evidence that the strategy learning route chosen by subjects, and thus the resulting generality/specificity of that strategy, can be predicted by computing the best strategy given the training set they experience. The framework that we utilized in this endeavor stands in contrast to previous approaches that have relied on somewhat loose (and often seemingly post-hoc) descriptions of task conditions that result in flexible and/or inflexible learning (e.g., whether the task is “easy” or “hard” [6]). Here we were able to make an explicit *a priori* determination as to which strategy would be most effective under what conditions and thus were able to make clear predictions regarding the behavioral outcomes that would be observed in our 4Traj and 20Traj groups. The framework also provides a satisfying and parsimonious explanation for the question of *why* subjects select a given learning route – they simply choose the route that maximizes performance on the given task.

In the context of a motion extrapolation task, we assessed the use of two different subject-adopted strategies: one that is model-based and one based on categorization-based stimulus-response mapping. When the training paradigm did not provide a reliable route to *bypassing* model-based extrapolation, subjects continued to use extrapolation and thus suffered no decrements in performance at post-test. Conversely, when training provided a viable opportunity to forego costly and error prone model-based predictions in favor of a simpler categorization-based mapping, subjects quickly transitioned to the less demanding approach. While this shift in tactic provided substantial benefits on the trained task itself (indeed, in our experiments 4Traj subjects increased their accuracy by approximately 60%), the fact that the categorization scheme was only truly appropriate for the training task conditions meant that there was a significant cost to performance when the conditions changed (i.e., at post-test). In essence, the mapping strategy was both highly efficient and highly inflexible - it allowed the subjects to perform the single trained task very well, but to do little else when the context changed.

Subjects' adoption of the categorization-based strategy revealed two additional insights about action selection. First, adoption of simpler, training-specific strategies did not occur at the expense of more general abilities. That is, when subjects were provided with feedback that the categorization strategy is not suitable for the task at post-test, they rapidly returned to their original model-based strategy. Therefore, task training adds to the toolbox, rather than replaces old ones, allowing individuals to choose the best tool for the immediate task. Second, use of the simpler categorization strategy may not actually be for the purposes of successful extrapolation more generally per se, but instead, for the purpose of minimizing costs associated with action planning. Not only do subjects continue to use the categorization scheme at post-test, they continue to use the same scheme at post-test for multiple extrapolation distances. That is, they use the strategy to bypass extrapolation altogether. This is the least computationally demanding strategy supported by these subjects' experience, as it never requires a forward predictive model. Thus, we additionally conclude that people exploit any shortcuts that may be revealed through training.

It is worth noting though that there is room for improvement in these *a priori* predictions. In particular, approximately 25% of the 4Traj subjects did not conform. Rather than continuing to utilize the categorization strategy during the post-test, they immediately switched back to using their predictive model. We could find no particular trend in the data to allow us to predict which subjects would be part of this 25%. Those that did switch back may have had other knowledge or made alternative inferences regarding the task context that we could not directly measure with the current paradigm. According to our overarching framework, however, we would predict that the cognitive cost associated with running the predictive model forward should be measurably less in these subjects. This is a hypothesis that remains to be tested in future work.

Furthermore, subjects in the 20Traj condition learned very little during the course of training. However, we expected the number of trials in 20Traj training to be insufficient to learn a 20-category strategy. This result is reasonable given that 20Traj subjects had only 20 trials training per trajectory, rather than the 100 trials per trajectory experienced by the 4Traj subjects. Moreover, given trajectory confusability, the error feedback in training contained little information that could impose changes on the model. Thus, our 4Traj and 20Traj training conditions were designed to create a scenario in which subjects could learn a simpler categorization model or stick with their original predictive model. Future work will be devoted to establishing conditions under which subjects will be expected to learn a new predictive model, or, more likely, improve an existing one, in order to benefit task performance. Such work will contribute further insight into the effects of training on action strategy learning and selection.

The distinction between flexible and inflexible learning has been made in many distinct areas of the learning literature [7]–[9], but without knowing the set of alternative strategies and the cost/benefit ratios of those various strategies in advance, it is difficult to predict when each will arise. Here, we show that by starting with an inherently predictive task, training conditions can be established that directly promote inflexible learning. Overall, the general outcome observed in 4Traj subject performance is consistent with the finding of inflexible (‘specific’) learning prevalent in the perceptual learning literature. That the majority of the 4Traj subjects were unable to adapt to the new trajectories at posttest despite achieving near ceiling performance during training, is analogous to typical reports of this field: substantial improvements in performance on the trained task, but no ability to perform new tasks (i.e., no transfer of learning) (see [10] for a review). For instance, subjects trained to discriminate whether a field of moving dots was moving just barely clockwise or counterclockwise from straight up will show a substantial improvement in accuracy through training. However, when they are subsequently asked to perform the same discrimination task around a new angle (e.g., straight left), no benefits of the training are observed [11]–[13]. Similarly inflexible learning has been observed for low-level visual features such as orientation, contrast, texture, and retinal location [14]–[18]. Not surprisingly, given the results reported here, the training conditions most commonly utilized throughout this literature are consistent with those that we have shown promote the use of a task specific strategy – namely large numbers of trials and very small stimulus sets. This same basic finding has been shown in other domains as well, such as motor and cognitive training. Similarly, the fact that only the subjects trained with a more variable stimulus set (i.e., the 20Traj subjects) retained the ability to generalize is also consistent with the examples in the literature of training paradigms that produce flexible (‘general’) learning [19]–[23]. In particular, these training experiments have commonly used highly variable stimulus sets, which prevent learning of specific strategies that are reliable.

Although our task does differ from these and other classic learning tasks in a number of potentially key ways, the parallels between our findings and those of other learning domains suggests important links can be made. We believe that our findings and framework offer insight into general principles of learning, beyond any specific learning context. For example, the notion of ‘overfitting’ (see [10] for a review) in perceptual learning can be operationalized as policy learning, as described above. Nevertheless, it will be left to future work to determine whether the framework we put forth here can directly account for the results in more ‘classic’ learning tasks.

Outside of the domain of perceptual learning, policy learning, or learning a direct mapping between a condition and a response, is implicated as a major hypothesis for habit-based learning. A similar prediction of habit-based dominance was put forward by [24]. In their work, the reliability of performance determines whether a model-based strategy is adopted versus a simpler model-free strategy. Other domains also describe the phenomenon of automaticity. For instance, when subjects are asked to simulate how they would move the steering wheel of a car to make a lane change to the left, most fail somewhat dramatically [25], moving the wheel to the left and then straightening (which would very quickly see them go off the left side of the road), rather than moving the wheel to the left and then back to the right. In other words, removing the input breaks the trained or automatic habitual response. In essence, these subjects can initiate a lane-change but lack a method of predicting what the next motor action should be and thus they cannot run this seemingly simple process forward for the two seconds that it would take to change lanes. Instead, subjects appear to rely entirely on a set of automatically executed mappings between cues in the environment and actions they should take (e.g., if the car is going off toward the left side of the road, turn the steering wheel back right). Similar automaticity is observed in other domains, such as in catching fly balls in baseball. This is a clear situation where one could, in principle, perform an extensive look ahead when the ball is struck. This would allow the fielder to run directly to the most likely landing location (which would be constantly updated as more and more of the trajectory was observed). However, in practice, human fielders appear to execute a series of automatic responses based on the flight of the ball (e.g., to run in such a way as to cancel the vertical acceleration of the image of the ball on the retina; [26]–[28]).

Our results also bear similarity to findings in the statistical learning literature. For example, Chalk et al [29] provide evidence that subjects develop biases to report perceived motion direction consistent with the motion distribution of the training stimuli. Our subjects similarly exhibit a change in their responses whereby the choices are consistent with the distribution of responses used during training. However, unlike previous authors who conclude that the statistical learning biases perception of the stimuli, we conclude that learning biases the responses made. This is supported by the fact that subjects immediately switch strategies when they are explicitly signaled that the task context has changed; that is, we have no evidence that our subjects perceive the stimulus differently, nor evidence to suggest that our subjects are using fundamentally different perceptual information in the categorization and the extrapolation cases.

In sum, our primary goal is to contribute new understanding about the mechanisms underlying training-based changes in task performance. Towards that end, we establish a new approach towards predicting and training the learning of different action strategies. We also provide results that span accounts from several literatures. Specifically, the categorization-based responses of our 4Traj subjects link these literatures, as the responses embody the characteristics of inflexible (non-generalizable) learning of model-free policies that map trajectories directly to specific responses, with little sensitivity to changes in context (i.e., significant failures once removed from the trained scenario). Finally, we provide clear evidence that learning is the direct result of the training paradigm, establishing perceptual learning as one potential future avenue of research. The importance of the goal of training must be reflected in the design of any training paradigm. If the goal is to produce a large amount of learning on only a specific task, with no need for generalization, then the best route is large numbers of training trials on only that task. However, if the goal is to produce learning that generalizes beyond the specifics of the training task, it is essential to ensure that the parameters of the task provide no feasible option to transition to a computationally simpler mapping strategy.

## Methods

### Ethics Statement

All research was carried out with approval by the University of Minnesota IRB, protocol number: 0201M16281 and Assurance of Compliance number: FWA00000312.

### Model Simulations

The goal of the model simulations was to assess the relative efficacy of a mapping strategy versus a predictive model strategy (given a human-like ability to estimate trajectory parameters such as curvature and orientation) as a function of the number of trajectory-response pairs that needed to be learned. More specifically, we wished to identify conditions that strongly favored one strategy over the other, which could then be used in an experiment with human subjects. We believe this provides a significant advance in the study of conditions that result in flexible versus rigid learning.

To evaluate the effectiveness of utilizing a predictive model on the task, we used a linear state space model, often termed a Kalman filter, a standard algorithm used in modeling trajectory estimation [30]–[33]. At each time step, the Kalman filter maintains an internal representation of the dot's state – its position, velocity, and acceleration. During the visible portion of the trajectory, the Kalman filter repeatedly runs through a cycle of prediction, observation, and correction. It applies its internal model to the dot's state to make a prediction about the dot's state at the next time step. Then it observes the actual position at the next time step (corrupted slightly by noise), and corrects its estimate based on this new observation for each time step where the dot is observable. When the dot hits the occluder, the Kalman filter continues its predictions through the duration of occlusion without observation/correction steps (see **Text S2** of the Supporting Information for further details). The total number of trials was set to 2000 and simulated for 4,8,12,16, and 20 trajectories.

To evaluate the effectiveness of learning mappings, we simulated Q-learning [34]–[36], a standard iterative learning approach used in the reinforcement learning literature to learn a response mapping (see **Text S1** of the Supporting Information for details). Briefly, the agent learns an action-value function that gives the expected value, in terms of performance of each response in a given state – the trajectory. Actions-values are learned via experience – for each trajectory, the agent makes a response and tabulates its performance feedback for this trajectory-response pair. The agent's “knowledge” is thus represented by a look-up table, Q, in which each row corresponds to a specific trajectory, and each column corresponds to one of the possible choice bins. We performed five separate Q-learning simulations. We simulated performance after learning under the same conditions as above (2000 trials each for training sets 4,8,12,16, & 20 trajectories). Because there are more repetitions of individual trajectories for small set sizes, mapping performance will degrade with training set size.

Relative model performance was quantified as the log ratio of percent correct choices. **Figure 2C** shows that the mapping strategy outperformed the predictive model strategy for training sets less than 10, after which the performance reversed. We chose to test subjects on set sizes of 4 and 20 as these conditions strongly favored one of the strategies.

### Human Behavior

#### Experiment 1

17 members of the University of Minnesota community participated in exchange for course credit or monetary compensation. Ten were randomly assigned to the 4Traj group, and seven to the 20Traj group. Four additional subjects participated in the 4Traj version of the experiment with a modified post-test task (details will be described below). All were unaware of the purpose of the study and had normal or corrected-to-normal vision.

The pre-test and post-test extrapolation stimuli consisted of displays similar to that depicted in **Figure 2A**, which contained a dot (0.25° radius) that traveled continuously along a circular arc. The space of possible trajectories consists of the curvature ranges: {+0.0016:+0.0079} & {−0.0016:−0.0079}, where curvature is defined as the inverse of the radius of the circle from which the trajectory was drawn, and orientation ranging between +60° and −60°. These ranges were chosen because they prevent “illegal” trajectories (i.e., ones that curve back towards the point of occlusion before reaching the curved edge of the occluder) as well as straight lines (curvature = 0). The dot traveled rightward along its trajectory of length 4° at a constant velocity of 12°/sec. When it reached the center of the screen, it disappeared behind the midpoint of the straight edge of a half-disk shaped occluder with radius 4°. On the opposite, curved edge of the occluder resided 20 bins demarcated by black straight lines and numbered in a clockwise fashion from top (−90°) to bottom (+90°). The lines flanking the subject's choice bin turned white at the end of each trial. The pre- and post-test trajectory set contained 320 paths chosen randomly from the full space of curvatures and orientations, with 80 coming from each quadrant (all four combination of +/− curvature, +/− orientation) – this ensured subjects did not develop specific expectations about the types of trajectories they might encounter during these sessions. Subjects viewed each moving dot stimulus and after the dot moved behind the occluder, they used the mouse to click one of the 20 bins indicating where they believed that the dot would reemerge from occlusion. No feedback was given so as to reduce learning effects during these test sessions. Subjects also participated in an extrapolation/generation task in which they drew the extrapolated trajectory through the occlusion region on the screen with the mouse. The display during drawing was similar to that during extrapolation except the bins were omitted. The block consisted of 80 trials: the transfer trajectory set (see below), presented four times each. Following pre-test subjects were randomly assigned to either the 4Traj or a 20Traj training group (see below).

The stimulus display during training was similar to that used during pre-test and post-test with the two key differences. First, training consisted of 5 blocks of 80 trials per block. Within these blocks either four trajectories were presented 20 times per block (in the 4Traj group) or 20 trajectories were each presented four times each (in the 20Traj group). The four trajectories presented to the 4Traj group were a subset of the twenty trajectories presented to the 20Traj group. Second, feedback was given after each trial both in the form of a color cue (if the subject was correct the lines around the chosen bin turned green, otherwise the lines around the chosen bin turned red) and in the form of the dot reemerging from occlusion (i.e., if the subject chosen the wrong bin they not only knew that they were incorrect, but they also knew what the correct choice would have been).

Interleaved among training blocks were no-feedback transfer blocks. Each transfer block consisted of 80 trials: 20 total trajectories consisting of the four from 4Traj training, four more from 20Traj training, and eight new trajectories from the overall parameter space were each presented four times.

In all experimental sessions, the stimuli were presented on a 19.5-in. monitor with 1280×1024 resolution with a vertical refresh rate of 75 Hz. Subjects viewed the display from a distance of 52 cm, such that one pixel corresponded to 0.032 degrees of visual angle (DVA). The stimuli were viewed under conditions of low ambient illumination with subjects' heads fixed by means of a chin rest.

#### Experiment 2

17 new members of the University of Minnesota community participated in the experiment, which comprised three sessions (pre-test, training, post-test) on consecutive days in exchange for course credit or monetary compensation. As in Experiment 1, ten were randomly assigned to the 4Traj group and seven to the 20Traj group. All were unaware of the purpose of the study and had normal or corrected-to-normal vision.

The pre-test and post-test extrapolation stimuli were similar to those of Experiment 1, however, three occluder radii were used: 2.8, 4, & 5.2 DVA. The mid-sized occluder corresponds to that used in Experiment 1. With the addition of the larger occluder, the space of possible trajectories is slightly reduced with the curvature ranges: {+0.0016:+0.0044} & {−0.0016:−0.0044}. This was to ensure once again that none of the trajectories, namely those with larger curvatures, circled back towards the point of occlusion before reaching the edge of all three occluders. Trajectory orientation continued to range between −60 and +60 deg.

The pre- and post-test sessions consisted of 600 trials each, 3 radii×200 trajectories selected from the full space. Unlike in Experiment 1, this trajectory set was specifically chosen so that 2/3 would reach a different bin for each radius while 1/3 did not need to meet this requirement. This resulted in all subjects receiving the identical trajectory sets, with randomized presentation.

The stimulus display during training was identical to that during the training session of Experiment 1, using only the mid-sized occluder. As before, training consisted of 5 blocks of 80 trials: either four trajectories (this time reemerging in bins 3,8,12,&18) were presented 20 times per block (4Traj) or 20 trajectories were each presented four times (20Traj), according to the group assignment. The four trajectories were a subset of the twenty trajectories.

Interleaved among training blocks were no-feedback transfer blocks. The stimulus display during the transfer blocks was identical to that used in pre- and post-test. Each block also consisted of 80 trials: 20 total trajectories consisting of the four from 4Traj training, plus 16 new trajectories from the parameter space, each presented four times.

All other methodological details were identical to Experiment 1.

## Supporting Information

Figure S1 Individual 4Traj subject performance during the no-feedback transfer portion of the training-transfer session. Note that each transfer block was preceded by an 80-trial training block with feedback (recall **Figure 2B** in the main text). By the end of the first block, many subjects already show a tendency to choose the four bins that are matched to the four training trajectories. This choice distribution is inconsistent with the true distribution of bins matched to the transfer trajectory set (set inset). However, some subjects never adopt this strategy (e.g., S9 & S10) and instead show extrapolation-like behavior during all no-feedback sessions (i.e., pre-test, transfer, and post-test). These subjects who do not adopt the four-bin selection strategy do, however, learn to properly choose the four bins during the feedback training trials.
(TIFF)

Figure S2 Transfer block performance broken down by trained and untrained trajectories for the two training groups. For the 4Traj-trained subjects, there is a significant advantage for trained trajectories versus untrained trajectories. This is due largely to the fact that the majority of these subjects continue to use the four trained bins for all trajectories during these blocks (see **Figure S1**). The 20Traj subjects also exhibit a small but non-significant advantage for trained trajectories. Generally, the 20Traj's group use of a prediction-based strategy provides them with a performance advantage in comparison with their 4Traj-trained counterparts who rely on a categorization-based strategy for untrained trajectories.
(TIFF)

Figure S3 Individual subject performance on the drawing task for all subjects who participated in Experiment 1 with the same format as Figure 4 in the main text. The set of trajectories presented to subjects during the drawing task was the same as the set of trajectories tested during the transfer blocks of the main bin choice task experiment (see **Figure 2B**).
(TIFF)

Figure S4 **A.** Pre- and posttest choice distributions for all seven individual 20Traj subjects. **B.** Pre- and posttest choice distributions for all ten individual 4Traj subjects plotted in the same format as in A in which the top row for each subset of subjects corresponds to pretest choices and the bottom row corresponds to the posttest choices.
(TIFF)

Figure S5 Pre- and post-test choice distributions for all four individual 4Traj subjects who received feedback halfway through post-test.
(TIFF)

Figure S6 Post-test choice distributions for all individual 4Traj subjects who participated in Experiment 2.
(TIFF)

Figure S7 Post-test choice distributions for all individual 20Traj subjects who participated in Experiment 2 in the same format as **Figure S6**.
(TIFF)

Text S1 Description of model-free (Q-learning) simulations.
(DOCX)

Text S2 Description of extrapolation model-based (Kalman filter) simulations.
(DOCX)

Text S3 Description of training-induced classification strategy simulation and data analysis.
(DOCX)
