# Supplementary figures and images for: Task-Specific Response Strategy Selection on the Basis of Recent Training Experience
Source: PLoS Comput Biol. 2014 Jan 2;10(1):e1003425. doi: 10.1371/journal.pcbi.1003425 (PMC3879094; doi:10.1371/journal.pcbi.1003425)

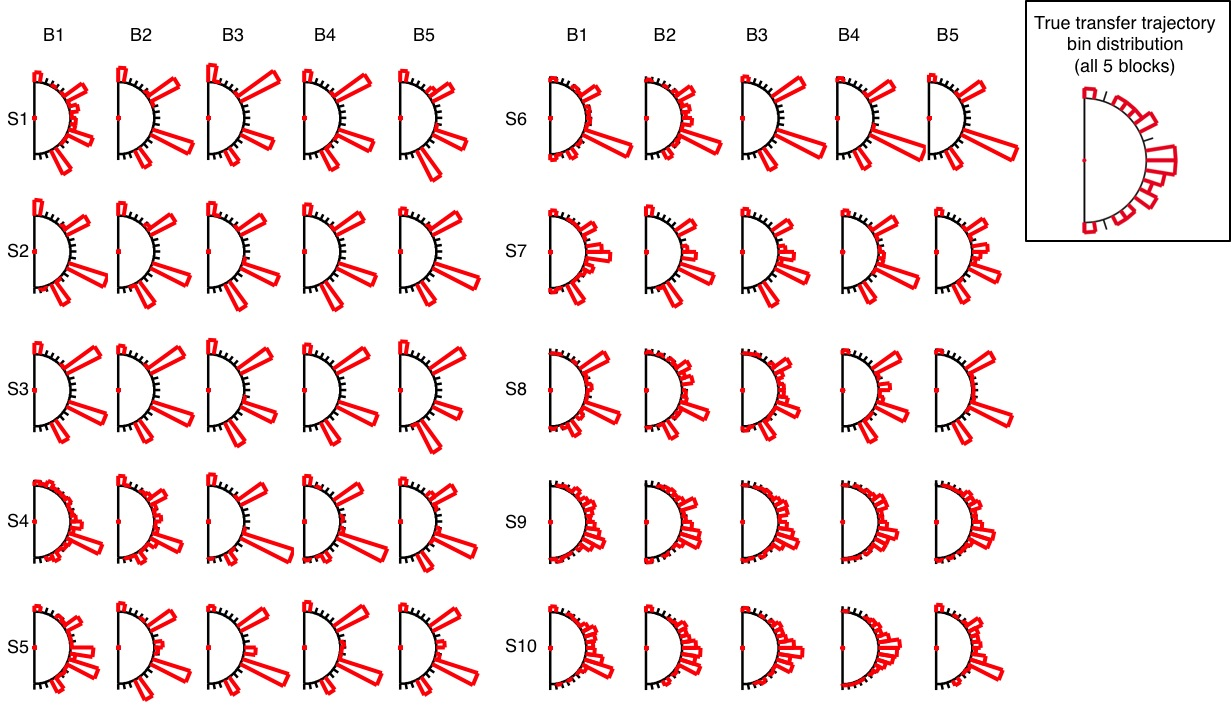

Supplement: Figure S1 — Individual 4Traj subject performance during the no-feedback transfer portion of the training-transfer session. Note that each transfer block was preceded by an 80-trial training block with feedback (recall Figure 2B in the main text). By the end of the first block, many subjects already show a tendency to choose the four bins that are matched to the four training trajectories. This choice distribution is inconsistent with the true distribution of bins matched to the transfer trajectory set (set inset). However, some subjects never adopt this strategy (e.g., S9 & S10) and instead show extrapolation-like behavior during all no-feedback sessions (i.e., pre-test, transfer, and post-test). These subjects who do not adopt the four-bin selection strategy do, however, learn to properly choose the four bins during the feedback training trials. (TIFF) [file pcbi.1003425.s001.tiff]

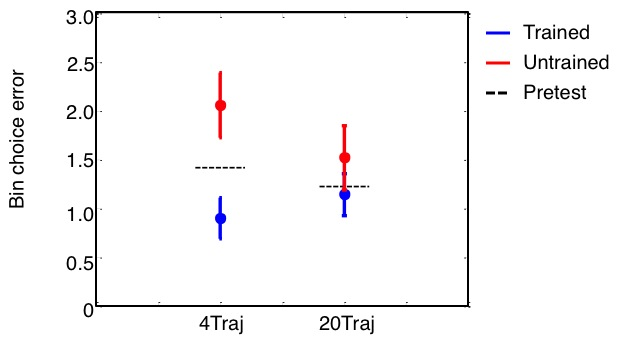

Supplement: Figure S2 — Transfer block performance broken down by trained and untrained trajectories for the two training groups. For the 4Traj-trained subjects, there is a significant advantage for trained trajectories versus untrained trajectories. This is due largely to the fact that the majority of these subjects continue to use the four trained bins for all trajectories during these blocks (see Figure S1). The 20Traj subjects also exhibit a small but non-significant advantage for trained trajectories. Generally, the 20Traj's group use of a prediction-based strategy provides them with a performance advantage in comparison with their 4Traj-trained counterparts who rely on a categorization-based strategy for untrained trajectories. (TIFF) [file pcbi.1003425.s002.tiff]

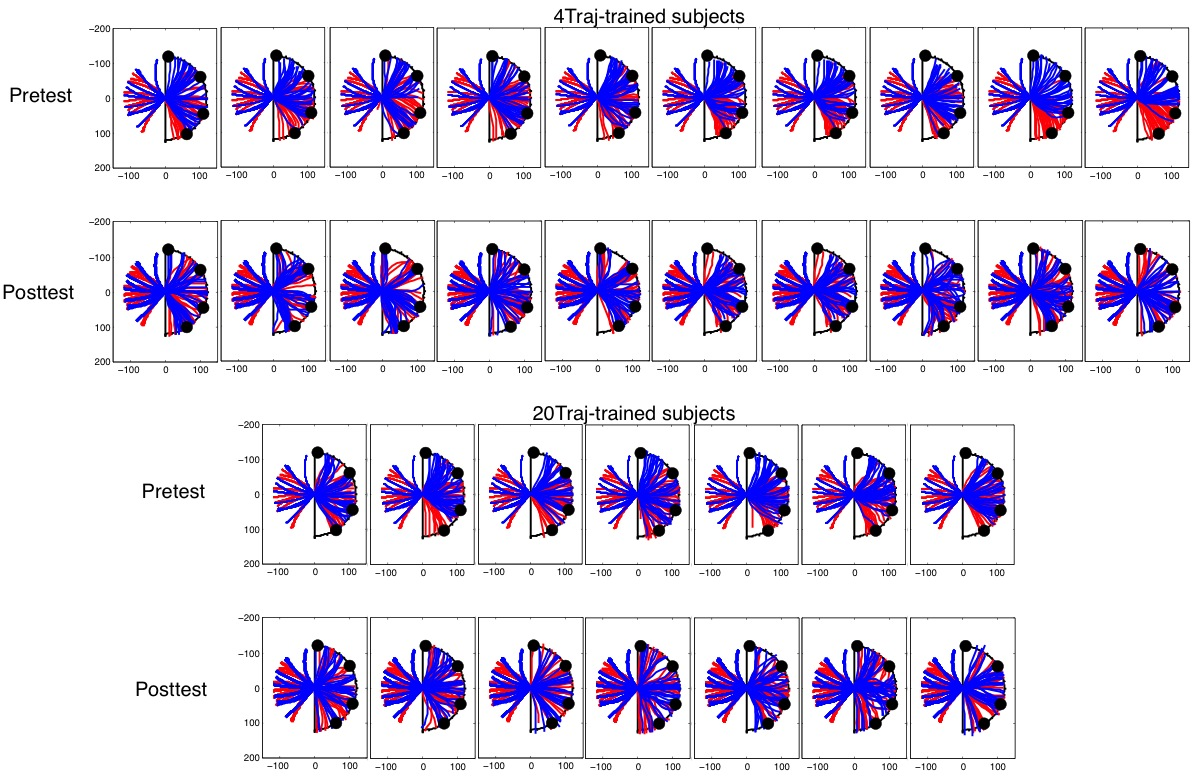

Supplement: Figure S3 — Individual subject performance on the drawing task for all subjects who participated in Experiment 1 with the same format as Figure 4 in the main text. The set of trajectories presented to subjects during the drawing task was the same as the set of trajectories tested during the transfer blocks of the main bin choice task experiment (see Figure 2B). (TIFF) [file pcbi.1003425.s003.tiff]

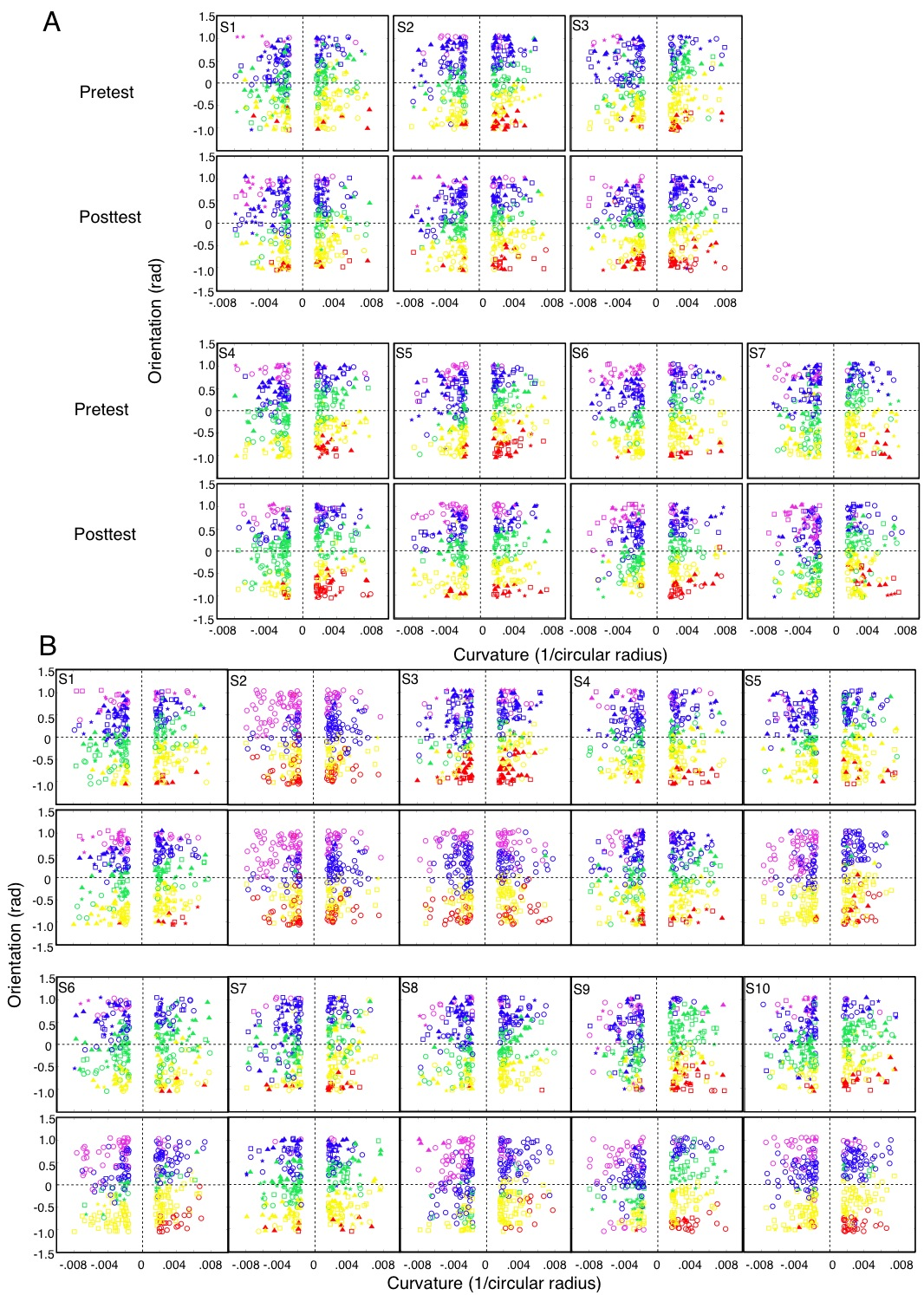

Supplement: Figure S4 — A. Pre- and posttest choice distributions for all seven individual 20Traj subjects. B. Pre- and posttest choice distributions for all ten individual 4Traj subjects plotted in the same format as in A in which the top row for each subset of subjects corresponds to pretest choices and the bottom row corresponds to the posttest choices. (TIFF) [file pcbi.1003425.s004.tiff]

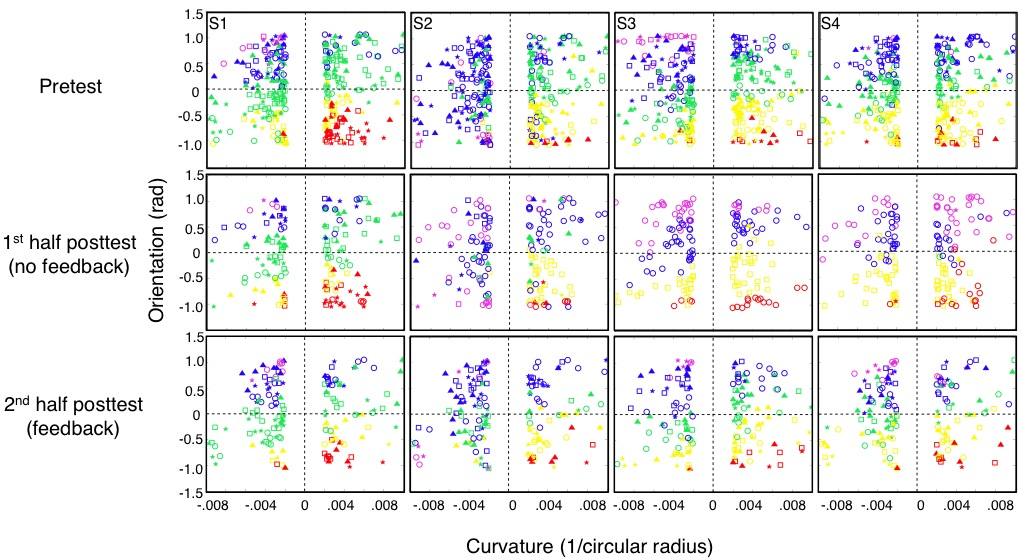

Supplement: Figure S5 — Pre- and post-test choice distributions for all four individual 4Traj subjects who received feedback halfway through post-test. (TIFF) [file pcbi.1003425.s005.tiff]

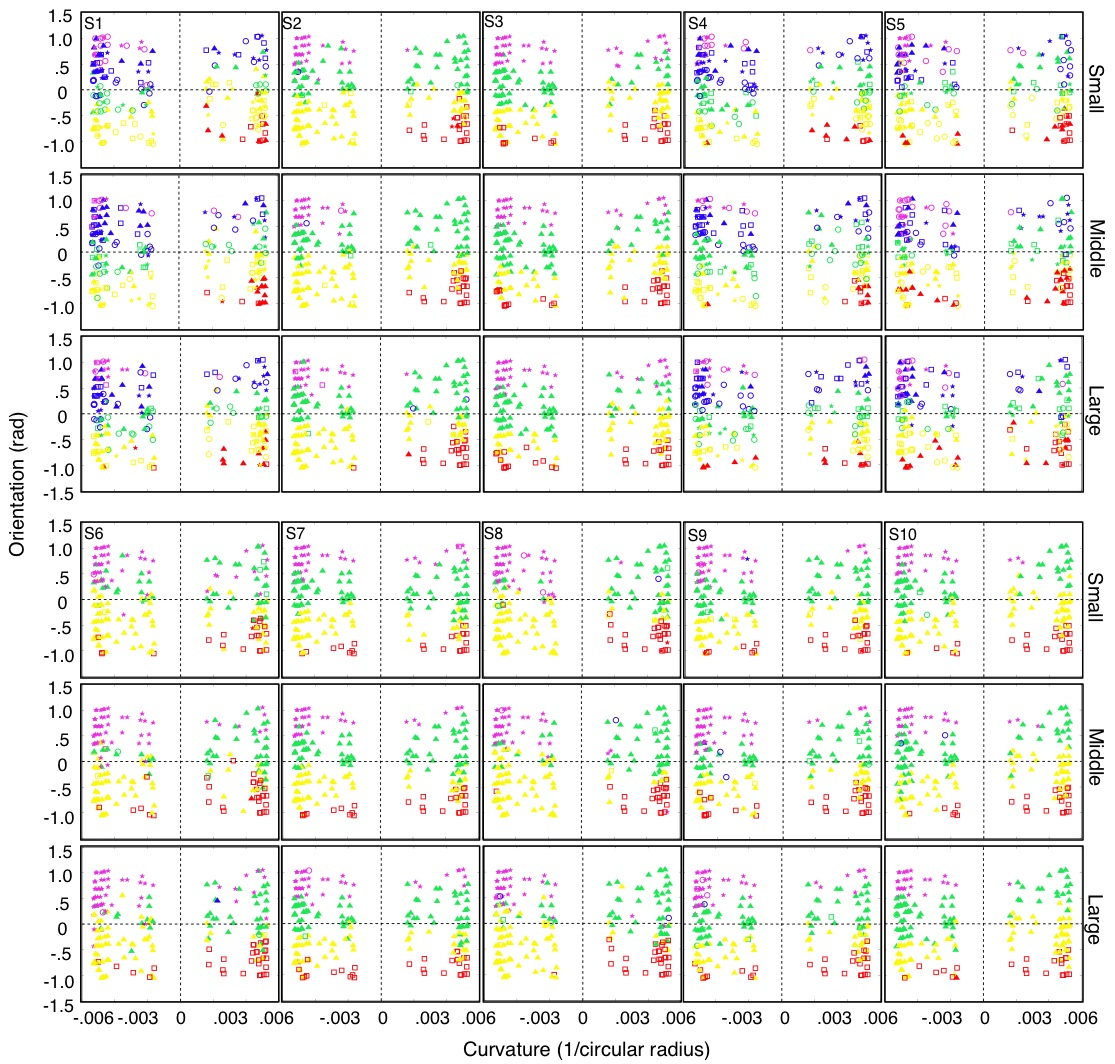

Supplement: Figure S6 — Post-test choice distributions for all individual 4Traj subjects who participated in Experiment 2. (TIFF) [file pcbi.1003425.s006.tiff]

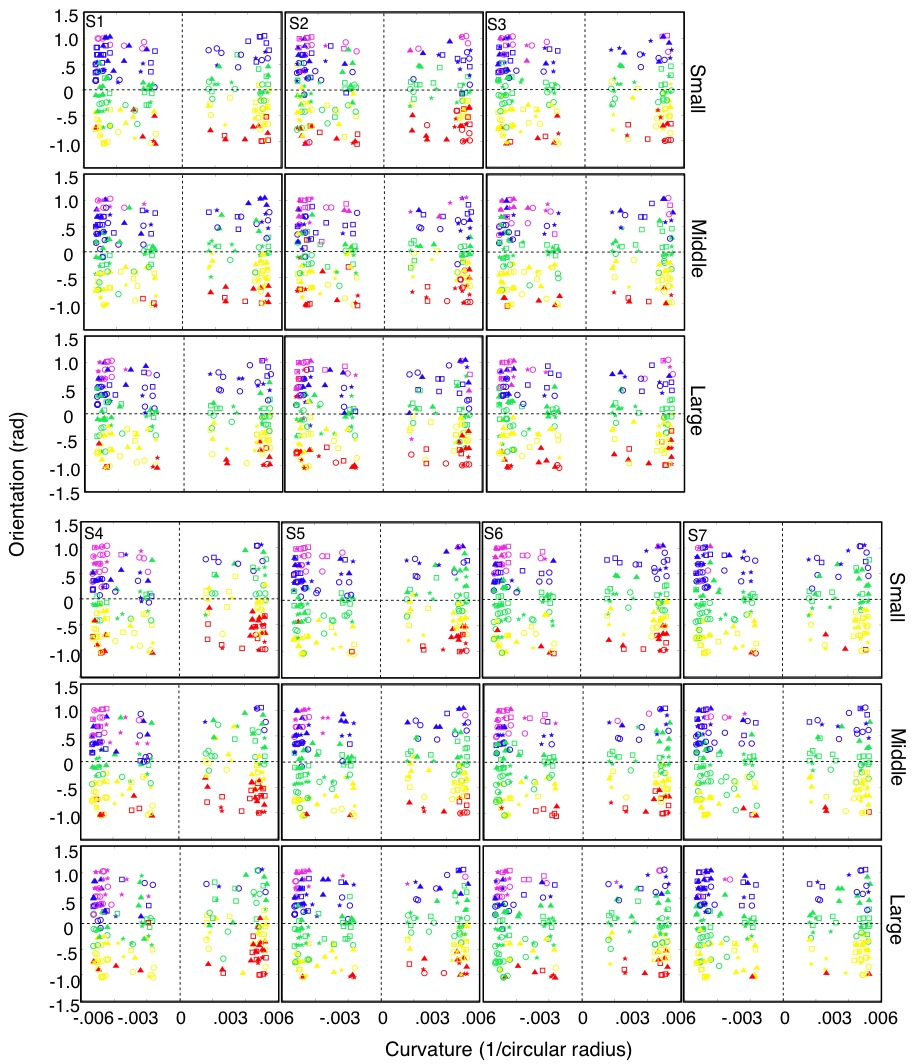

Supplement: Figure S7 — Post-test choice distributions for all individual 20Traj subjects who participated in Experiment 2 in the same format as Figure S6. (TIFF) [file pcbi.1003425.s007.tiff]
