# Supplementary material for: Task-Specific Response Strategy Selection on the Basis of Recent Training Experience
Source: PLoS Comput Biol. 2014 Jan 2;10(1):e1003425. doi: 10.1371/journal.pcbi.1003425 (PMC3879094; doi:10.1371/journal.pcbi.1003425)
Supplement: Text S1 — Description of model-free (Q-learning) simulations. (DOCX) [file pcbi.1003425.s008.docx]

**Simulated model-free performance (Q-learning)**

A model-free strategy that determines bin choices in the trajectory extrapolation task would rely on learned mappings between specific trajectories and numbered bins with experience in the task. We employ Q-learning [34; 35], a standard iterative learning approach used in the reinforcement learning literature to simulate such behavior. The agent (learner) learns an [action-value function](http://en.wikipedia.org/w/index.php?title=Action-value_function&action=edit&redlink=1) that gives the expected utility of taking a given action in a given state. Once a particular action dominates, i.e., is expected to have the greatest probability of reward, it will be chosen every time the corresponding state is visited, achieving a fixed policy. The model-free aspect derives from the ability of Q-learning to compare and learn the expected utility of the available actions independent of a model of the environment.

The agent’s “knowledge” is represented by a look-up table, *Q*, in which each row, *s*, in the context of the trajectory extrapolation task, corresponds to a specific trajectory, and each column, *a*, corresponds to one of the possible choice bins (see **Figure 2C** in the main text). The entry for each combination of trajectory and bin corresponds to the probability of choosing the bin. Initially, the simulated subject has no knowledge regarding trajectory-endpoint pairings, which is embodied in a uniform distribution on choices from which one is randomly selected—i.e., all bins are equally likely[[1]](#footnote-1). On each “trial”, an input, encoded by a state number (here, a trajectory number), is provided. Bin selection is made by probability matching via a ‘soft max’ decision rule (e.g., [36]), which tends to choose the option with the maximum value (or highest probability of success), but is ‘softened’ by both the value of the competing choices as well as randomness (noise) added to the decision rule.

Once a bin is chosen, feedback is provided: ‘0’ if the choice was incorrect or ‘1’ if correct. The feedback is used to update the *Q* matrix for future action selection according to Equation (S1):

$Q(s_{t},a_{t})\leftarrow Q(s_{t},a_{t})+\alpha(R(s_{t+1},a_{t+1})+\gamma\max_{a_{t+1}}(Q(s_{t+1},a_{t+1}))-Q(s_{t},a_{t}))$ (S1)

where $Q(s_{t},a{}_{t})$ is the old value before the choice was made, $\alpha$is the learning rate, which we fixed to 0.8 for all “trials”, $R(s_{t+1},a_{t+1})$is the reward value (0 or 1) associated with the choice $a_{t}$, $\gamma$ is the discount factor which we also fixed to 0.8, and $\max_{a_{t+1}}Q(s_{t+1},a_{t+1})$ is the predicted future “best” action when the state is next visited.

The learning rate determines how strongly new information will be weighted against the current information. The $\alpha=0.8$ learning rate weights new information more heavily. The discount factor determines how “greedy” the agent in its reward maximization. The $\gamma=0.8$ discount factor favors maximizing long-term reward.

In order to predict when a transition may occur between model-free and model-based behavior and to prepare the right-hand plot in **Figure 2C** in the main text, we performed five separate Q-learning simulations. The total number of trials was set to 2000 and the state (trajectory) space consisted of 4,8,12,16, and 20 trajectories. This ensured an equal number of trials per trajectory in each trajectory set condition, and an equal amount of “experience” overall. The trajectories were randomly selected from the full space of allowable trajectories (see the description of the stimuli in the main text). Although their precise parameters did not affect the Q-learning algorithm, they do affect the simulated model-based performance (see next section). Percent correct was simply computed as the percentage of correct choices (number of trials in which the reward = 1) in the 2000 trials.

1. Indeed, human subjects do have some belief about where the trajectory will reemerge and thus do not initially make bin choices at random. This effectively reduces the space of choice bins to a possible subset at the start. However, because trajectory estimation beliefs and performance are variable across subjects, we chose to simulate the model-free strategy with no initial knowledge to sidestep these individual differences. This strategic decision is expected to result in more conservative estimates of the number of trials needed for the model-free strategy to converge on fixed policies—i.e., humans will likely learn the mappings with fewer trials. [↑](#footnote-ref-1)
