# Supplementary material for: Task-Specific Response Strategy Selection on the Basis of Recent Training Experience
Source: PLoS Comput Biol. 2014 Jan 2;10(1):e1003425. doi: 10.1371/journal.pcbi.1003425 (PMC3879094; doi:10.1371/journal.pcbi.1003425)
Supplement: Text S2 — Description of extrapolation model-based (Kalman filter) simulations. (DOCX) [file pcbi.1003425.s009.docx]

**Simulated performance using model-based trajectory extrapolation (Kalman filter)**

A model-based strategy that determines bin choices in the trajectory extrapolation task would rely on statistical estimation of the trajectory parameters in making predictions about the object’s motion according to an internal dynamics model. We employ the Kalman filter, a standard algorithm used in modeling this trajectory estimation (e.g., 30-33).

Given a choice of polynomial (dynamics) model and available visual information, the Kalman filter recursively estimates the state of the moving dot (i.e., the trajectory parameters) at each time step using an internal representation:

$s_{t}=As_{t-1}+\omega_{t-1}$ (S2)

The equation represents the linear extrapolation (prediction) from the internal representation of the object $s_{t-1}$ at time *t-1* to the predicted state at the next moment in time, $s_{t}$. The transition matrix *A* defines and represents the specific extrapolation strategy by encoding assumptions about the object’s dynamics process.

Here, we focus on local polynomial expansions of 2^nd^ degree (i.e. locally quadratic). Locally quadratic extrapolation is defined by the *A* matrix:

$A=\left[\begin{matrix} 1 & 0 & \Delta t & 0 & 0 & 0 \\ 0 & 1 & 0 & \Delta t & 0 & 0 \\ 0 & 0 & 1 & 0 & \Delta t & 0 \\ 0 & 0 & 0 & 1 & 0 & \Delta t \\ 0 & 0 & 0 & 0 & 1 & 0 \\ 0 & 0 & 0 & 0 & 0 & 1 \end{matrix}\right]$ (S3)

which involves state estimates of position, velocity, and acceleration in x and y, respectively, and captures the curvature involved in the trajectory of an object undergoing constant acceleration in a gravitational field and has been previously found to underlie static contour extrapolation through occlusion [4-5].

The term $\omega_{t-1}$ in Equation (S2) represents the process uncertainty (noise) with $p(\omega)\sim N(0,Q)$ with *Q* being a covariance matrix. The process noise represents the subject’s own estimate of model error.

At each time step, the current state, $\hat{\text{s}}_{\text{t-1}}\text{ }$, and error covariance, $\text{P}_{\text{t-1}}$, are projected to the next time step, via the *prediction* step, using the following formulas:

$\hat{\text{s}}_{\text{t}}^{\text{-}}\text{ = A }\hat{\text{s}}_{\text{t-1}}\text{ }$ (S4)

$\text{P}_{\text{t}}^{\text{-}}\text{ = AP}_{\text{t-1}}\text{A}^{\text{T}}\text{+Q}$ (S5)

Then the *observation* of the true object state, $z_{t}$ occurs, following Equation (S6):

$z_{t}=Hs_{t}+v_{t}$ (S6)

The equation represents the linear relationship between the object’s true state, $s_{t}$ and the observable parameters of that state (i.e., from the visible trajectory), defined by the matrix $H$. We assume that subjects’ measurements are dominated by the velocity of the object’s path. As such, the *H* matrix is defined as:

$H=\left[\begin{matrix} 0 & 0 & 1 & 0 & 0 & 0 \\ 0 & 0 & 0 & 1 & 0 & 0 \end{matrix}\right]$ (S7)

The observations are corrupted by sensory measurement uncertainty. The term $v_{t}$ in Equation (S6) represents this sensory uncertainty with *p(v)~N(0,R*) with *R* being a covariance matrix. This term represents the perceptual sensitivity of the subject to the observable trajectory.

An estimate of the error between the prediction and the observation is computed, along with an estimate of the error covariance:

$\text{e}_{\text{t}}\text{= z}_{\text{t}}\text{ -H}_{\text{t}}\hat{s}_{t}^{-}$ (S8)

$\text{S}_{\text{t}}\text{ = HP}_{\text{t}}^{\text{-}}\text{H}^{\text{T}}\text{+R}$ (S9)

The error covariance is used to compute the Kalman gain, $\text{K}_{\text{t}}$, which acts as a blending factor, that weights the model’s estimates against the observations:

$\text{K}_{\text{t}}=\text{P}_{t}^{-}H^{T}S_{t}^{-1}$ (S10)

The Kalman gain serves to minimize the *a posteriori* error covariance in the computations of the *a posteriori* state and covariance estimates, $\hat{\text{s}}_{\text{t}}$ and $\text{P}_{\text{t}}$, respectively, using the following formulas:

$\hat{\text{s}}_{\text{t}}\text{ = }\hat{\text{s}}_{\text{t}}^{\text{-}}\text{ + K}_{\text{t}}\text{e}_{\text{t}}$ (S11)

(S12)

Keeping in mind that the latter portion of the motion trajectory is occluded in our task, no measurements can be taken during this period. The prediction continues, however, using the process above, with the only omission being the $\text{K}_{\text{t}}\text{e}_{\text{t}}$ term from the state update equation (Eq. S11).

In modeling human performance, we provide the Kalman filter with x,y coordinates of the same trajectories that we provide the Q-learning agent (note that the precise parameters do not factor into the Q-learning agent’s performance). The y-coordinates are given a small amount of random Gaussian jitter to simulate sensory noise. The process and sensory measurement uncertainty (Eqns (S2) & (S6)) parameters are set to levels consistent with human performance in preliminary work. The Kalman filter runs through the prediction-observation cycle for the duration of the visible trajectory, and then continues its predictions through the duration of occlusion without feedback (observation). Once the Kalman filter has extrapolated the visible trajectory to the occluder’s edge, the process is terminated. We compute the polar angle of the extrapolated trajectory’s endpoint and determine which bin the trajectory is thus expected to reemerge within—this is recorded as the Kalman filter’s choice for that “trial”. Percent correct is once again computed as the percentage of correct choices (number of trials in which the choice bin = the “true” bin) in the 2000 trials.
