# Supplementary material for: Task-Specific Response Strategy Selection on the Basis of Recent Training Experience
Source: PLoS Comput Biol. 2014 Jan 2;10(1):e1003425. doi: 10.1371/journal.pcbi.1003425 (PMC3879094; doi:10.1371/journal.pcbi.1003425)
Supplement: Text S3 — Description of training-induced classification strategy simulation and data analysis. (DOCX) [file pcbi.1003425.s010.docx]

**Simulated performance using a training-induced classification strategy and evaluation of subject choice strategies**

We began by computing the probability of choosing each of the 20 bins on any given trial under the full extrapolation model (20 categories) and the four-category classifier model based on the four bins used during training. For the full model, we assume no preference over bins, thus all bins are equally likely to be chosen with probability 1/20=0.05. For the four-category classifier, we allowed for the possibility that subjects may choose non-training bins (especially at pretest) by including a fifth “lapse” category whose probability was set to a modest 2%. Thus, a non-training bin would have a probability of 0.02/16=0.0013, while a training bin would have a probability of (1-0.02)/4=0.245.

For each trajectory encountered by a given subject in a given session[[1]](#footnote-1) (i.e., pretest or posttest), we computed its “fit” to each bin as the multivariate Gaussian probability given the mean trajectory curvature and orientation and covariance associated with each bin. These probabilities were then weighted by the probability of choosing each bin.

Once we obtained these probability distributions for bin choice, we evaluated the probability of each subject’s actual bin choices under the two models for the session (n=320 trajectories). The relative weight of the four-category classifier, $w_{classifier}$, in accounting for the subject’s data was finally computed according to Equation S13:

$w_{classifier}=\frac{\frac{1}{2}\sum_{t=1}^{320} \log(p(b|M_{classifier}))}{\frac{1}{2}\sum_{t=1}^{320} \log(p(b|M_{classifier}))+\frac{1}{2}\sum_{t=1}^{320} \log(p(b|M_{extrapolation}))}$ (S13)

where $p(b|M_{classifier})$ is the probability of a subject’s bin choice for trial trajectory *t* under the four-category classifier model and $p(b|M_{extrapolation})$ is the probability of a subject’s bin choice for trial trajectory *t* under the full extrapolation model. The results were used to compute carry out a Bayesian model comparison of the four-category classifier model performance relative to the full model:

$K=\frac{p(B|M_{classifier})}{p(B|M_{extrapolation})}$ (S14)

where $p(B|M_{classifier})$is the probability of the subject’s full set of bin choices under the four-category classifier model and $p(b|M_{extrapolation})$is the probability of the subject’s full set of bin choices under the full extrapolation model.

The log of these ratios are plotted in **Figure 7** in the main text. We interpret the results according to conventional standards: positive K values support M_classifier_ and negative values support M_extrapolation_. Values larger than 100 are considered to be decisive evidence for the particular model. In our case, all values reach the decisive criterion in either direction.

1. We of course did not expect subject performance to be consistent with a classification strategy that uses the four training bins at pretest prior to training. Evaluating classification at pretest was done simply to provide a baseline degree of classification (and relative performance between the classifier and extrapolation models) for comparison at posttest. [↑](#footnote-ref-1)
